# Entropy generation and dissipative heat transfer analysis of mixed convective hydromagnetic flow of a Casson nanofluid with thermal radiation and Hall current

**DOI:** 10.1038/s41598-021-83124-0

**Published:** 2021-02-16

**Authors:** A. Sahoo, R. Nandkeolyar

**Affiliations:** Department of Mathematics, National Institute of Technology Jamshedpur, Jamshedpur, 831014 India

**Keywords:** Applied mathematics, Nanoscience and technology

## Abstract

The present article provides a detailed analysis of entropy generation on the unsteady three-dimensional incompressible and electrically conducting magnetohydrodynamic flow of a Casson nanofluid under the influence of mixed convection, radiation, viscous dissipation, Brownian motion, Ohmic heating, thermophoresis and heat generation. At first, similarity transformation is used to transform the governing nonlinear coupled partial differential equations into nonlinear coupled ordinary differential equations, and then the resulting highly nonlinear coupled ordinary differential equations are numerically solved by the utilization of spectral quasi-linearization method. Moreover, the effects of pertinent flow parameters on velocity distribution, temperature distribution, concentration distribution, entropy generation and Bejan number are depicted prominently through various graphs and tables. It can be analyzed from the graphs that the Casson parameter acts as an assisting parameter towards the temperature distribution in the absence of viscous and Joule dissipations, while it has an adverse effect on temperature under the impacts of viscous and Joule dissipations. On the contrary, entropy generation increases significantly for larger Brinkman number, diffusive variable and concentration ratio parameter, whereas the reverse effects of these parameters on Bejan number are examined. Apart from this, the numerical values of some physical quantities such as skin friction coefficients in *x* and *z* directions, local Nusselt number and Sherwood number for the variation of the values of pertinent parameters are displayed in tabular forms. A quadratic multiple regression analysis for these physical quantities has also been carried out to improve the present model’s effectiveness in various industrial and engineering areas. Furthermore, an appropriate agreement is obtained on comparing the present results with previously published results.

## Introduction

Nanofluids are generated by colloidal suspensions of nanosized particles in the base fluid. The nanoparticles are usually made of metals, oxides, carbides, or carbon nanotubes. For example, the base fluids are taken as water, ethylene, glycol, oil and many others. It is observed to have various important useful properties of nanofluid such as the increment of the heat transfer and the stretching rate of nanofluid. The increment in the effectiveness and performance of coolant is required in various areas such as electronics, power production, vehicle, engineering and industrial system etc. Generally, the nanofluid coolant is used to increase the quality of aerodynamics designs. Recently, nanofluid is applied in various cases, such as aerodynamics power engineering, heat exchanger, cooling of transformer, chemical separation devices, solar water heating, micropamps and drug recovery system. For huge requirements, the researchers are motivated to investigate the coolant, which has a high performance. So the researchers want to enhance the thermal conductivity of traditional fluids like ethyline glycol, water, oil etc. The thermal conductivity of ordinary base fluids is very low, and it is necessary to enhance the thermal conductivity of base fluids. The Suspension of nanoparticles in the base fluid improves the thermal conductivity and convective heat transfer. Initially, Choi^1^ accepted this idea and introduced an innovative new type of nanofluids, which expresses a high thermal conductivity. Eastman et al.^2^ established a nanofluid containing copper nanometer-sized particles dispersed in ethylene glycol, and the nanofluid’s thermal conductivity was larger than any other pure ethylene glycol. Khan and Pop^3^ suggested an innovative mathematical model on the steady flow and thermal behaviour of nanofluid flowing over a linearly stretching sheet. Seth et al.^4^ executed a comprehensive study on an attractive mathematical model containing the MHD mixed convection stagnation point flow of micropolar nanofluid.

The study on flow over a stretching sheet is significantly important for using its application in many engineering and industrial sectors. Its fascinated applications are utilized in the production of plastic and rubber sheets, metalworking process such as hot rolling, aerodynamic extrusion of plastic sheets, melt spinning as a metal forming technique, elastic polymer substance and production of emollient, paints, production of glass-fibre etc. Crane^5^ executed an investigation on the solution of boundary layer equation of Newtonian fluid over a stretching plate. Generally, Crane’s suggested model of the linearly stretching plate is not used in many industrial sectors. So Researchers find an interest for investigating the various aspects of the stretching rate. Remembering the vast applications of the stretching rate, Fang et al.^6^ investigated boundary layer flow over a stretching sheet with a power law velocity assuming the variable thickness of the sheet. The influences of different controlling parameters and different solution branches on the velocity and shear stress distributions were prominently illustrated. Rana and Bhargava^7^ analyzed flow and heat transfer of a nanofluid over a nonlinearly stretching sheet. Here the combined effects of Brownian motion and thermophoresis were prominently discussed. Kameswaran et al.^8^ examined Hydromagnetic nanofluid flow due to a stretching or shrinking sheet by taking viscous dissipation and chemical radiation effects into account. Here the external magnetic field significantly affects the nanofluid flow over a stretching sheet and controls the boundary layer of nanofluid. The impacts of magnetic field and viscous dissipation on the wall heat and mass transfer rates were highlighted significantly. Some other relevant and innovative investigations under different conditions are discussed by several authors^9–12^.

The flow of an electrically conducting fluid in the presence of magnetic field is utilized in many engineering devices, such as MHD propulsion system, plasma confinement, liquid-metal cooling of nuclear reactors, electromagnetic pumps, MHD generators etc. The strong magnetic field generates a resistive Lorentz force, which controls the flow. In heat transfer processes, for getting the remarkable outcomes of the product, the rate of cooling can be controlled. Under the influence of the externally applied magnetic field, the cooling rate of liquid is controlled. The researchers emphasize the study on magnetohydrodynamic fluid flow due to its immense potentials for using in various engineering and industrial problems. For the requirements of the new aspects of the investigation, the researchers move towards analyzing the magnetohydrodynamic fluid flow. Recalling the remarkable applications of this work, Pavlov^13^ was the first to develope an interesting model regarding the incompressible magnetohydrodynamic flow of a viscous fluid past a stretching surface. Sheikholeslami et al.^14^ displayed a keen interest to address the numerical simulation of MHD nanofluid flow and heat transfer between two parallel plates in a rotating system by taking the effect of viscous dissipation into account. They discussed various important results including the nature of the magnitude of the skin friction coefficient and Nusselt number against the disparate values of pertinent parameters. They showed that magnetic parameter and rotation parameter had favourable effects on the magnitude of the skin friction coefficient, but the adverse effects of both of these parameters on Nusselt number were visualized. Khan and Makinde^15^ studied MHD laminar boundary layer flow of an electrically conducting water-based nanofluid containing gyrotatic microorganisms along a convectively heated stretching sheet. They incorporated the convective boundary layer condition. Hsiao^16^ initiated the model regarding micropolar nanofluid flow towards a stretching sheet with the multimedia feature in the presence of MHD and viscous dissipation effects by taking Brownian motion and thermophoretic effect into account. Contributions on the topic of MHD flow of the electrically conducting fluid under different conditions are depicted in the articles^17–19^.

It is an established fact that the flow of an electrically conducting fluid under the impact of a magnetic field produces a transverse flow because of the effect of Hall current, which rises due to the strong intensity of the magnetic field. The Hall effect has the potential to deal with many real life problems, and has a great importance to signify different flow features within the flow field. As this context, for its remarkable applications in various cases, the researchers find a keen interest to analyze theoretically and graphically about the impact of Hall current on the MHD flow of the viscous, incompressible and electrically conducting fluid. Maleque and Sattar^20^ studied the effects of variable properties along with the effects of suction/injection and Hall current on a steady MHD convective flow generated by an infinite rotating porous disk. They inferred that Hall parameter *m* had an amazing effect on the radial and axial velocity profiles. They noticed that increasing the values of $m(>2.0)$ resulted in diminishing the radial and axial velocity profiles. Keeping the earlier research of works on the effect of Hall current in mind, Khan et al.^21^ tried to derive the exact analytical solutions for the MHD flows of an Oldroyd-B fluid through a porous space in the presence of the effect of Hall current. Some of the recent research works about this phenomena are executed by several authors^22–26^.

For the improvement of the realistic fluid flow problems, it is necessary for the researchers to move towards analyzing the time dependent models. Firstly, Wang^27^ tried to execute the unsteady-state problem. The pioneering attempt to find a fluid film on an accelerating stretching surface was done by Wang. He introduced a similarity transformation to turn the Navier–Stokes equations into the nonlinear ordinary differential equations. Attia^28^ presented an interesting model for analyzing the impact of the external uniform magnetic field on the unsteady flow of an incompressible, viscous and electrically conducting fluid over an infinite rotating porous disk. Freidoonimehr et al.^29^ initiated a fascinating model to investigate the effect of the unsteady MHD laminar free convective flow of nanofluid over a porous vertical surface. They analyzed the effect of various parameters like magnetic parameter, unsteadiness parameter, buoyancy parameter etc. on velocity and temperature distribution. They examined that unsteadiness parameter was highly responsible for decreasing skin friction coefficient, whereas the reverse effect of it on the rate of mass transfer was observed evidently. Some of the relevant and effective research works are done by several authors^30–32^.

The investigation of non-Newtonian fluid attracts the researchers very much due to its huge applications in industrial and engineering areas. In 1995, Casson established a fluid flow model along with the flow of non-Newtonian liquids. Casson fluid is one type of nanofluid, and it has a great significance in various cases. Recently, the Casson fluid flow model becomes meaningful for its fascinated application in human life. The examples of Casson fluid are honey, Chilly sauce, jelly, blood etc. The Casson fluid flow model has a remarkable requirement in modern science. Casson fluid displays the properties of yield stress. However, when yield stress becomes large, Casson fluid turns into a Newtonian fluid. If yield stress is less than shear stress, Casson fluid starts move. Taking care of it Eldabe and Salwa^33^ made a first attempt to investigate the heat transfer of steady MHD non-Newtonian Casson fluid flow between two co-axial cylinders. Many years had passed to improve the investigation of this work. Nadeem et al.^34^ discussed the influence of the externally applied magnetic field on the Casson fluid flow in two lateral directions past a porous and linear stretching sheet. They presented the interesting results against the variation of Casson flow parameter as well as other fluid flow parameters. Recalling huge requirements of Casson fluid in real life, Prashu and Nandkeolyar^35^ introduced a mathematical model to get the interesting results about the influence of unsteady three dimensional incompressible and electrically conducting magnetohydrodynamic flow of Casson fluid over the stretching sheet under the combined effects of radiative heat transfer and Hall current. Various relevant and useful investigations are presented by several authors^36–39^.

Heat transfer system is significantly performed by thermal radiation. The effect of thermal radiation finds the potential for using in many industrial and engineering applications, such as electrical power generation, nuclear energy plants, astrophysical flows, space vehicles, solar systems, gas production etc. In the present investigation, our motive is to develope various models which depict the impact of radiative heat transfer on the magnetohydrodynamic fluid flow under different conditions. Mbeledogu and Ogulu^40^ established an amazing mathematical model regarding heat and mass transfer of an unsteady MHD flow of a rotating fluid past over a vertical porous flat plate with taking radiative heat transfer and natural convection into account. They estimated that increasing the values of the Prandtl number and the radiative parameter diminished the temperature of fluid within the boundary layer. Ansari et al.^41^ investigated the flow of non-Newtonian viscoelastic nanofluid over a linearly stretching sheet under the impact of the uniform magnetic field and radiative nonlinear heat transfer. The remarkable and innovative studies about this present phenomena are illustrated in the articles^42–45^.

In a thermodynamic system, the entropy generation is the amount of entropy which is created generally during irreversible processes by means of heat flow through a thermal resistance, fluid flow through a flow resistance, diffusion, Joule heating, friction between solid surfaces, fluid viscosity within a system etc. According to the second law of thermodynamics, the total entropy of the system remains unchanged during a reversible process. On the other hand,over a surface, when nanofluid flows are passing through several irreversible processes, such as diffusion, friction between the layers of fluid due to viscosity, thermal resistance, flow resistance, Joule heating etc., then the increment in the total entropy of the system can be observed. It is well known that entropy generation has a crucial role to diminish the required sources of energy of the system. In order to get better efficiency and performance in most engineering and industrial applications, the key concern of the researchers is to lessen the entropy generation. Taking care of this, initially, Bejan^46^ tried to investigate the entropy generation in a convective heat transfer process. Shit et al.^47^ scrutinized a mathematical model to analyze entropy generation on unsteady two-dimensional magnetohydrodynamic flow of nanofluid over an exponentially stretching surface in a porous medium under the influence of thermal radiation. This research work was extended by Shit and Mandal^48^. They treated Buongiorno’s model to investigate entropy generation on unsteady magnetohydrodynamic flow of Casson nanofluid over a stretching vertical plate under the influence of thermal radiation. Their investigation suggests that Casson parameter increases entropy generation sharply, while thermal radiation increases it closer to the plate. In this context, some of the relevant and remarkable investigations are described in the articles^49–55^.

In quantum statistical mechanics, the idea of entropy was promoted by John von Neumann. Consequently, the entropy called as “von Neumann entropy” is actually an extension of the classical Gibbs entropy concepts to the field of quantum mechanics, and is generally defined as follows $S=-k_{B}Tr\left( \rho \log \rho \right)$ where $k_{B}$ is Boltzmann constant, and $\rho$ is the density matrix. Researchers are interested to investigate the fruitful aspects of various applications of quantum statistical mechanics for its immense requirements in a wide number of industrial and engineering processes. Taking care of it, Wang et al.^56^ explored the quantized quasi-two-dimensional Bose–Einstein condensates with spatially modulated nonlinearity in the harmonic potential. Liang et al.^57^ initiated a family of exact solutions of the one-dimensional nonlinear Schrödinger equation for analyzing the dynamics of a bright soliton in Bose–Einstein condensates with the time-dependent interatomic interaction in an expulsive parabolic potential. Wen et al.^58^ displayed the matter rogue wave in Bose–Einstein condensates with attractive interatomic interaction analytically and numerically. Chen et al.^59^ combined the cellular dynamical mean-field theory with the continuous time quantum Monte Carlo method for getting the rich phase diagrams in the Hubbard model on the triangular kagome lattice as a function of interaction, temperature, and asymmetry. Moreover, in the field of quantum mechanics, Abliz et al.^60^ analyzed the entanglement control in an anisotropic two-qubit Heisenberg XYZ model in the presence of the external magnetic fields. Hu et al.^61^ described a brief explanation on the basis of the necessary and sufficient conditions for local creation of quantum correlation. Qi et al.^62^ represented a real physical system containing the non-Abelian Josephson impact between two $F =2$ spinor Bose–Einstein condensates with double optical traps. Ji et al.^63^ proposed an optical system describing the photonic Josephson effects in two weakly linked microcavities with ultracold two-level atoms. Furthermore, Ji et al.^64^ introduced an optical system to let a direct experimental observation of the quantum magnetic correlated dynamics of the polarized light.

So far as the researchers are investigating the impact of unsteady three-dimensional magnetohydrodynamic flow of Casson nanofluid over a stretching sheet for its remarkable requirement in engineering and industrial applications. No one has discussed about the unsteady three-dimensional magnetohydrodynamic flow of Casson nanofluid over the stretching surface in the presence of radiative heat transfer and mixed convection with taking viscous dissipation, Brownian motion, Ohmic heating, thermophoretic effect, heat generation and Hall current into account. In this present article,the main objective of the authors is to represent a mathematical model containing the unsteady three-dimensional incompressible and electrically conducting magnetohydrodynamic flow of Casson nanofluid over a stretching sheet in a vertical direction under the influence of radiative heat transfer, Hall current, Mixed Convection, Viscous and Joule dissipations. Similarity transformations are utilized to turn the nonlinear partial differential equations into nonlinear ordinary differential equations. The obtained nonlinear coupled ordinary differential equations are numerically solved by using spectral quasi-linearization method $\left(\text{SQLM}\right)$. In this present article, the impacts of the variation of pertinent parameters such as Casson liquid parameter, Hall current parameter, magnetic parameter, unsteadiness parameter, radiation parameter, Brownian motion parameter, thermophoretic parameter, heat generation parameter, mixed convection parameter, Eckert number on the distribution of velocity, temperature and concentration are analyzed significantly. A brief description about entropy generation, and the impacts of various pertinent flow parameters on the entropy generation rate and Bejan number are displayed significantly.

## Mathematical formulation

**Figure 1:**
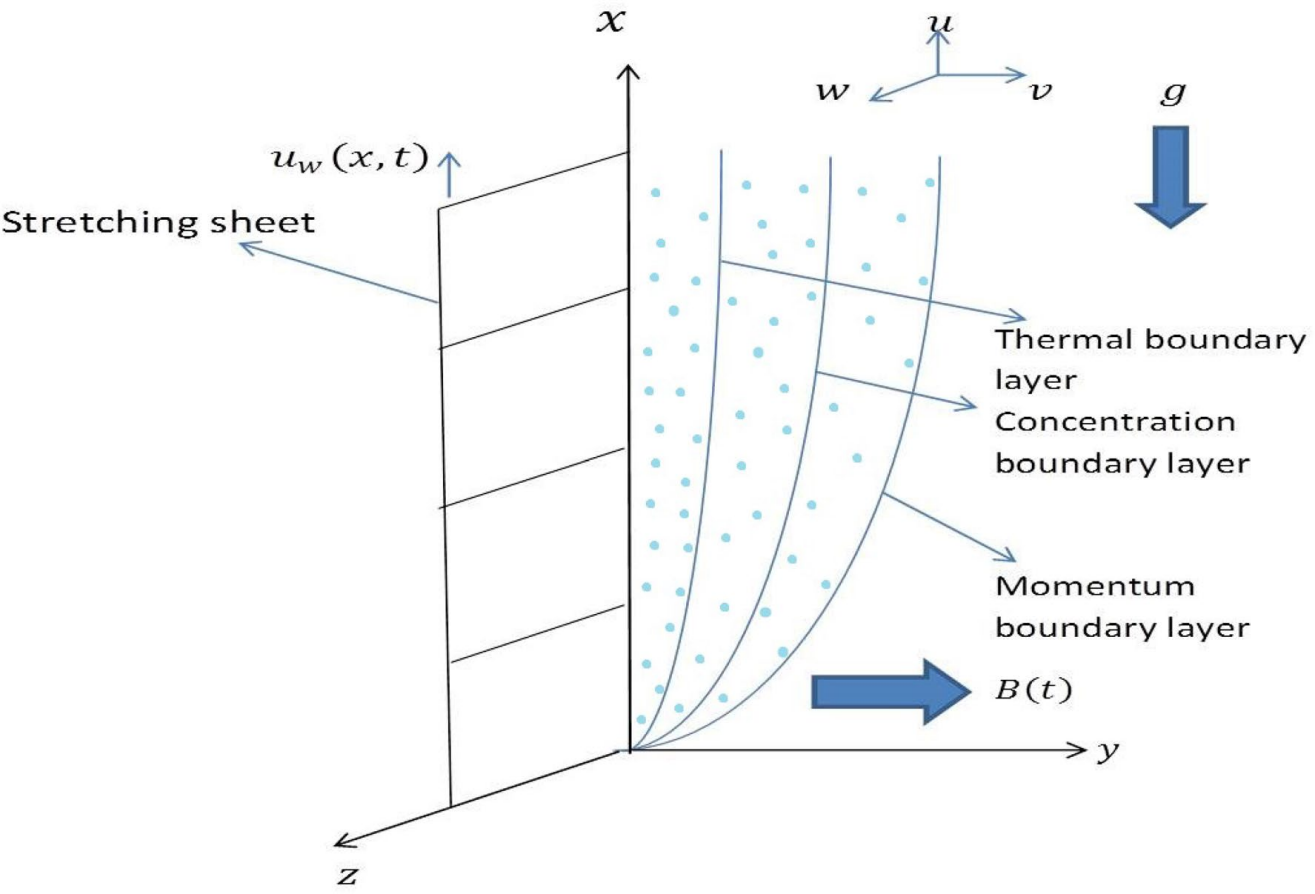
The schematic diagram of the physical problem.

We consider the unsteady three-dimensional flow of an incompressible, homogeneous, electrically conducting Casson nanofluid past a vertical stretching sheet in the presence of an external magnetic field. We assume that *x*-axis is along the sheet in the vertically upward direction, and *y*-axis is normal to the sheet. It is assumed that the nanofluid is occupies the region $y\geqslant 0$. We assume that the nanoparticle and base fluid are in thermal equilibrium, and the chemical reaction between them is neglected. It is assumed that, except the density in buoyancy force, the thermophysical properties of nanofluid remain constant. The Boussinesq approximation is taken into account so that the density variation obtained by concentration or temperature difference is neglected except in case of buoyancy force. The time-dependent velocity of the sheet in *x*-direction is assumed to be $u=u_{w}(x,t)$. The nanofluid is viscous and electrically conducting. The external time-dependent magnetic field *B*(*t*) is applied in the positive *y*-direction, which is normal to the surface of the sheet. The geometry of the problem is presented in Fig. 1. We also assume the magnetic Reynolds number to be very small ($Re_{m}\leqslant 1$) so that the induced magnetic field is neglected as compared to the applied one. The intensity of the applied magnetic field is so strong that the Hall current is generated in the flow field. The symbols $T=T_{w}$ and $T=T_{\infty }$ denote the constant temperature of the fluid at the sheet’s surface and in the free stream, respectively. $C_{w}$ is the nanoparticle fraction concentration at the sheet, and $C_{\infty }$ is the ambient concentration. The heat transfer phenomenon is also influenced by radiation, viscous dissipation, Brownian motion, Ohmic heating, thermophoretic effect, and heat generation.

The rheological equation of the Casson fluid is given as follows:

1 $$\begin{aligned} \tau _{ij}= {\left\{ \begin{array}{ll} 2\left( \mu _{B}+\frac{p_{y}}{\sqrt{2\pi }}\right) e_{ij}, &{}\quad \pi >\pi _{c}\\ 2\left( \mu _{B}+\frac{p_{y}}{\sqrt{2\pi _{c}}}\right) e_{ij}, &{}\quad \pi < \pi _{c}\\ \end{array}\right. } \end{aligned}$$

where $e_{ij}$ is the $(i,j){\text {th}}$ component of the deformation rate, $\mu _{B}$ is the plastic dynamic viscosity of the non-Newtonian fluid, $p_{y}$ is the yield stress of the fluid, $\pi =e_{ij}e_{ij}$ is the product of the component of the deformation rate with itself and $\pi _{c}$ denotes a critical value of this product based on the non-Newtonian model. Here $\beta =\frac{\mu _{B}\sqrt{2\pi _{c}}}{p_{y}}$ is the Casson parameter.

Using the above assumptions, the governing boundary layer equations i.e. continuity, momentum, energy and concentration equations can be expressed, respectively, as

2 $$\begin{aligned} \frac{\partial {u}}{\partial {x}}+\frac{\partial {v}}{\partial {y}}+\frac{\partial {w}}{\partial {z}}=0 \end{aligned}$$

3 $$\begin{aligned} \frac{\partial {u}}{\partial {t}}+u\frac{\partial {u}}{\partial {x}}+v\frac{\partial {u}}{\partial {y}}+w\frac{\partial {u}}{\partial {z}}=\nu _{nf}\left( 1+\frac{1}{\beta }\right) \frac{\partial ^2{u}}{\partial {y^2}}-\frac{\sigma B^2(t)}{\rho _{nf}(1+m^2)}\left( u+mw\right) +g\beta _t\left( T-T_{\infty }\right) \end{aligned}$$

4 $$\begin{aligned} \frac{\partial {w}}{\partial {t}}+u\frac{\partial {w}}{\partial {x}}+v\frac{\partial {w}}{\partial {y}}+w\frac{\partial {w}}{\partial {z}}=\nu _{nf}\left( 1+\frac{1}{\beta }\right) \frac{\partial ^2{w}}{\partial {y^2}}+\frac{\sigma B^2(t)}{\rho _{nf}(1+m^2)}\left( mu-w\right) \end{aligned}$$

5 $$\begin{aligned}&\frac{\partial {T}}{\partial {t}}+u\frac{\partial {T}}{\partial {x}}+v\frac{\partial {T}}{\partial {y}}+w\frac{\partial {T}}{\partial {z}}=\frac{\kappa _{nf}}{\left( \rho c_p\right) _{nf}}\frac{\partial ^2{T}}{\partial {y^2}}-\frac{1}{\left( \rho c_p\right) _{nf}}\frac{\partial {q_{r}}}{\partial {y}}\nonumber \\&\quad +\,\frac{\left( \rho c_p\right) _{np}}{\left( \rho c_p\right) _{nf}}\left[ D_{B}\frac{\partial {C}}{\partial {y}}\frac{\partial {T}}{\partial {y}}+\frac{D_T}{T_{\infty }}\left( \frac{\partial {T}}{\partial {y}}\right) ^2\right] +\frac{\mu }{(\rho c_p)_{nf}}\left( 1+\frac{1}{\beta }\right) \left[ u_{y}^{2}+w_{y}^{2}\right] \nonumber \\&\quad +\,\frac{Q(t)(T-T_{\infty })}{(\rho c_p)_{nf}}+\frac{\sigma B^{2}(t)}{(\rho c_p)_{nf}(1+m^{2})}\left[ u^2+w^2\right] \end{aligned}$$

6 $$\begin{aligned} \frac{\partial {C}}{\partial {t}}+u\frac{\partial {C}}{\partial {x}}+v\frac{\partial {C}}{\partial {y}}+w\frac{\partial {C}}{\partial {z}}=D_{B}\frac{\partial ^2{C}}{\partial {y^2}}+\frac{D_T}{T_{\infty }}\frac{\partial ^2{T}}{\partial {y^2}} \end{aligned}$$

The suitable boundary conditions are defined as

$$\begin{aligned} \left. \begin{array}{ll} \text {At}\; y=0{:}\; u=u_{w},\quad v=0,\quad w=0,\quad T=T_{w},\quad C=C_{w}\\ \text {As}\; y\rightarrow \infty {:}\; u\rightarrow 0,\quad w\rightarrow 0,\quad T\rightarrow T_{\infty },\quad C\rightarrow C_{\infty }; \end{array}\right\} \end{aligned}$$

where $u_{w}$ is the stretching velocity in x direction. Let $u_{w}(x,t)=\frac{ax}{1-\gamma t}$ and the time dependent magnetic field is taken as $B\left( t\right) =B_0\left( 1-\gamma t\right) ^{-1/2}$. Here *a* and $\gamma$ are constants. Further, in the above equations, *u*, *v* and *w* are the components of velocity in *x*, *y* and *z* directions, respectively. t is time variable, $\beta$ is the Casson fluid parameter, $\sigma$ denotes electrical conductivity, $\nu _{nf}$ is the nanofluid’s kinematic viscosity, *m* is Hall current parameter, T is the temperature of fluid within the boundary layer, C denotes nanoparticle concentration within boundary layer region, $\kappa$ denotes thermal conductivity of the nanofluid, $\left( \rho c_p\right) _{nf}$ denotes the heat capacity of nanofluid, $\left( \rho c_p\right) _{np}$ denotes nanoparticle heat capacity, $D_{B}$ is coefficient of Brownian diffusion, $D_T$ is coefficient of thermophoretic diffusion, and the radiative heat flux is denoted by $q_{r}$.

Here optically thick fluid is taken, and the radiative heat flux vector is defined with the help of the Rosseland approximation as follows

7 $$\begin{aligned} q_{r}=-\frac{4\sigma ^*}{3\alpha ^*}\frac{\partial {T^4}}{\partial {y}} \end{aligned}$$

where $\sigma ^*$ is Stefen–Boltzmann constant and $\alpha ^*$ is the coefficient of Rosseland mean absorption. Following Pantokratoras and Fang^65^, the radiative heat flux is simplified as

8 $$\begin{aligned} q_{r}=-\frac{16\sigma ^*}{3\alpha ^*}T^3\frac{\partial {T}}{\partial {y}} \end{aligned}$$

The present mathematical problem is executed by using radiative heat flux given in (8).

For simplifying the present mathematical model, the similarity transformations^66,67^ are generated as

$$\begin{aligned} \left. \begin{array}{ll} \eta =y\sqrt{\frac{a}{\nu \left( 1-\gamma t\right) }},\quad u=\frac{ax}{\left( 1-\gamma t\right) }f^{'}\left( \eta \right) ,\quad v=-\sqrt{\frac{a\nu }{\left( 1-\gamma t\right) }}f\left( \eta \right) ,\\ w=\frac{ax}{\left( 1-\gamma t\right) }g\left( \eta \right) ,\quad \theta \left( \eta \right) =\frac{T-T_{\infty }}{T_{w}-T_{\infty }},\quad \phi \left( \eta \right) =\frac{C-C_{\infty }}{C_{w}-C_{\infty }} \end{array}\right\} \end{aligned}$$

Utilizing the similarity transformations, the present governing equations turns into nonlinear coupled ordinary differential equations, which are presented as follows

9 $$\begin{aligned} \left( 1+\frac{1}{\beta }\right) f^{'''}+ff^{''}-f^{'2}-A\left( f^{'}+\frac{\eta }{2}f^{''}\right) -\frac{M}{1+m^2}\left( f^{'}+mg\right) +\lambda \theta =0 \end{aligned}$$

10 $$\begin{aligned} \left( 1+\frac{1}{\beta }\right) g^{''}-gf^{'}+fg^{'}-A\left( g+\frac{\eta }{2}g^{'}\right) +\frac{M}{\left( 1+m^2\right) }\left( mf^{'}-g\right) =0 \end{aligned}$$

11 $$\begin{aligned}&\left( 1+\frac{4}{3}Nr(1+(tr-1)\theta )^3\right) \theta ^{''}+4Nr(tr-1) (1+(tr-1)\theta )^2\theta ^{'2}-PrA\frac{\eta }{2}\theta ^{'}\nonumber \\&\quad +\,Pr\left( f\theta ^{'}+\alpha \theta \right) +Pr\left( Nb\phi ^{'}\theta ^{'} +Nt\theta ^{'2}\right) +PrEc\left( 1+\frac{1}{\beta }\right) \left( f^{''2}+g^{'2}\right) \nonumber \\&\quad +\,\frac{PrEcM}{(1+m^2)}\left( f^{'2}+g^{2}\right) =0 \end{aligned}$$

12 $$\begin{aligned} \phi ^{''}+\frac{Nt}{Nb}\theta ^{''}+Sc f\phi ^{'}-\frac{A}{2}\phi ^{'}\eta Sc=0 \end{aligned}$$

The boundary conditions reduce to the following form:

13 $$\begin{aligned} f^{'}=1,\quad f=0,\quad g=0,\quad \theta =1, \quad \phi =1\quad \text { at} \quad \eta =0 \end{aligned}$$

14 $$\begin{aligned} f^{'}\rightarrow 0, \quad g\rightarrow 0,\quad \theta \rightarrow 0, \quad \phi \rightarrow 0\quad as \quad \eta \rightarrow \infty \end{aligned}$$

where non-dimensional parameters are defined as

$$\begin{aligned}&{{\rm Unsteadiness}}\,{{\rm parameter}}\,A=\frac{\gamma }{a},\, {{\rm Magnetic}}\,{{\rm Parameter}}\, M=\frac{\sigma B_0^2}{\rho _{nf} a},\, {{\rm Radiation}}\,{{\rm parameter}}\,Nr=\frac{4\sigma ^{*}T_{\infty }^{3}}{\kappa _{nf} \alpha ^{*}},\\& {{\rm Prandtl}}\,{{\rm number}}\,Pr=\frac{\left( \mu c_p\right) _{nf}}{\kappa _{nf}},\, {{\rm Temperature}}\,{{\rm ratio}}\,{{\rm parameter}}\,tr=\frac{T_{w}}{T_{\infty }},\, {{\rm Schmidt}}\,{{\rm number}}\,Sc=\frac{\nu _{nf}}{D_{B}},\\ &{{\rm Brownian}}\,{{\rm motion}}\,{{\rm parameter}\,Nb=\frac{\left( \rho c_p\right) _{np}D_{B}\left( C_{w}-C_{\infty }\right) }{\left( \rho c_p\right) _{nf}\nu _{nf}},}\\& {{\rm Thermophoretic}}\,{{\rm parameter}}\,Nt=\frac{\left( \rho c_p\right) _{np}D_T\left( T_{w}-T_{\infty }\right) }{\left( \rho c_p\right) _{nf}\nu _{nf} T_{\infty }},\\& {{\rm Eckert}}\,{{\rm number}}\,Ec=\frac{u_{w}^2}{c_p(T_{w}-T_{\infty })},\\ &{{\rm Local}}\,{{\rm Reynolds}}\,{{\rm number}}\,Re_x=\frac{u_{w}x}{\nu _{nf}},\, {{\rm Local}}\,{{\rm Grashof}}\,{{\rm number}}\,Gr_x=\frac{g\beta _t(T_{w}-T_{\infty })x^3}{\nu _{nf}^2},\\ &{{\rm Mixed}}\,{{\rm Convection}}\,{{\rm parameter}}\,\lambda =\frac{Gr_x}{Re_x^2},\, {{\rm Heat}}\,{{\rm generation}}\,{{\rm parameter}}\,\alpha =\frac{Q_0}{a(\rho c_p)_{nf}}.\end{aligned}$$

## Quantities of physical interest

For engineering interest, the significant physical quantities, such as skin friction coefficients in *x* and *z* directions $C_{fx},\; C_{fz}$, the local Nusselt number (rate of heat transfer)$Nu_x$ and Sherwood number(rate of mass transfer)$Sh_x$ are defined as

15 $$\begin{aligned} C_{fx}=\frac{\tau _{wx}}{\rho u_{w}^{2}},\quad C_{fz}=\frac{\tau _{wz}}{\rho u_{w}^{2}},\quad Nu_{x}=\frac{xq_{w}}{\kappa _{nf}(T_{w}-T_{\infty })},\quad Sh_x=\frac{xJ_{w}}{D_{B}(C_{w}-C_{\infty })}. \end{aligned}$$

where the shear-stress components $\tau _{wx},\;\tau _{wz},\; \text {heat flux}\; q_{w}\;\text {and}\; \text {mass flux}\; J_{w}$ at the surface are defined as

16 $$\begin{aligned} \left. \begin{array}{ll} \tau _{wx}=\mu _{nf}\left( 1+\frac{1}{\beta }\right) \left( \frac{\partial {u}}{\partial {y}}\right) _{y=0},\quad \tau _{wz}=\mu _{nf}\left( 1+\frac{1}{\beta }\right) \left( \frac{\partial {w}}{\partial {y}}\right) _{y=0},\\ q_{w}=\left( -\kappa _{nf}\left( \frac{\partial {T}}{\partial {y}}\right) +q_{r}\right) _{y=0} ,\quad J_{w}=\left. -D_{B}\frac{\partial {C}}{\partial {y}}\right| _{y=0} \end{array}\right\} \end{aligned}$$

The present physical quantities in non-dimensional form are defined as

17 $$\begin{aligned} \left. \begin{array}{ll} C_{fx}Re_{x}^{\frac{1}{2}}=\left( 1+\frac{1}{\beta }\right) f^{''}(0),\quad C_{fz}Re_{x}^{\frac{1}{2}}=\left( 1+\frac{1}{\beta }\right) g^{'}(0), \\ Nu_{x}Re_{x}^{\frac{-1}{2}}=-\left[ 1+\frac{4}{3}Nr\left( 1+(tr-1)\theta (0)\right) ^3\right] \theta ^{'}(0),\quad Sh_{x}Re_{x}^{\frac{-1}{2}}=-\phi ^{'}(0). \end{array}\right\} \end{aligned}$$

where $q_{r}$ is the radiative heat flux and $Re_x=\frac{xu_{w}}{\nu _{nf}}$ is the local Reynolds number.

## Solution methodology

The obtained ordinary differential Eqs. (9)–(12) along with the boundary conditions defined in (13) and (14) are numerically solved by using spectral quasi-linearization method (SQLM). This method is utilized to linearize the nonlinear terms of the transformed ordinary differential equations with the help of the one term Taylor series approximation about the previous iteration, say r. Following the framework of SQLM, the resulting iterative scheme is presented as

18 $$\begin{aligned} a_{113,r}f_{r+1}^{'''}+a{112,r}f_{r+1}^{''}+a_{110,r}f_{r+1}+a_{110,r}f_{r+1}+a_{120,r}g_{r+1}=R_{1,r} \end{aligned}$$

19 $$\begin{aligned} a_{222,r}g^{''}_{r+1}+a_{221,r}g^{'}_{r+1}+a_{220,r}g_{r+1}+a_{211,r}f^{'}_{r+1}+a_{210,r}f_{r+1}=R_{2,r} \end{aligned}$$

20 $$\begin{aligned} a_{332,r}\theta ^{''}_{r+1}+a_{331,r}\theta ^{'}_{r+1}+a_{330,r}\theta _{r+1}+a_{310,r}f_{r+1}+a_{341,r}\phi ^{'}_{r+1}=R_{3,r} \end{aligned}$$

21 $$\begin{aligned} \phi ^{''}_{r+1}+a_{441,r}\phi ^{'}_{r+1}+a_{432,r}\theta ^{''}_{r+1}+a_{410,r}f_{r+1}=R_{4,r} \end{aligned}$$

The boundary conditions in iterative form are derived as

$$\begin{aligned} f_{r+1}^{'}=1, \quad f_{r+1}=0, \quad g_{r+1}=0, \theta _{r+1}=1, \quad \phi _{r+1}=1\quad at\quad \eta =0 \end{aligned}$$

and

$$\begin{aligned} f_{r+1}^{'}\rightarrow 0, \quad g_{r+1}\rightarrow 0,\quad \theta _{r+1}\rightarrow 0,\quad \phi _{r+1}\rightarrow 0\quad as\quad \eta \rightarrow \infty \end{aligned}$$

For starting the iterative scheme, the initial approximations, which satisfy the boundary conditions are assumed as $f_0=1- e^{-\eta },\quad g_0=0,\quad \theta _0=e^{-\eta },\quad \phi _0=e^{-\eta }$

For solving the linearized and decoupled equations (18)–(21) numerically, a well known method, namely the Chebyshev spectral collocation method, is used. The method uses the Chebyshev polynomials defined in $[-1,1]$ to discretize the computational domain. For this reason, the physical region $\left[ 0,\infty \right)$ is truncated to a domain $\left[ 0,L_{\infty }\right]$. Then the domain $\left[ 0,L_{\infty }\right]$ is transformed to the interval $\left[ -1,1\right]$ by utilizing the following linear transformation

$$\begin{aligned} \eta =\dfrac{L_{\infty }(\zeta +1)}{2},\quad -1\leqslant \zeta \leqslant 1. \end{aligned}$$

where $L_{\infty }$ is called scaling parameter, which is large but a finite number. It is chosen to present the behaviour of the flow properties outside the boundary layer region. Let P be the number of Gauss Lobatto collocation points. The Gauss Lobatto collocation points utilized to discretize the domain [− 1, 1] are defined as

$$\begin{aligned} \zeta _i=\cos \dfrac{\pi i}{P},\quad i=0,1,2\dots P \end{aligned}$$

At these P collocation points, the functions $F_{j},\; G_{j},\; \Theta _{j}\;\text { and}\; \Phi _{j}$ for $j\geqslant 1$ are approximated with the help of *k*th Chebyshev polynomial $\left( T_{k}^{*}\right)$ as follows

22 $$\begin{aligned} F_{j}\left( \zeta \right) \approx \sum _{k=0}^{P}F_{j}\left( \zeta _{k}\right) T_{k}^{*}\left( \zeta \right) \end{aligned}$$

23 $$\begin{aligned} G_{j}\left( \zeta \right) \approx \sum _{k=0}^{P}G_{j}\left( \zeta _{k}\right) T_{k}^{*}\left( \zeta \right) \end{aligned}$$

24 $$\begin{aligned} \Theta _{j}\left( \zeta \right) \approx \sum _{k=0}^{P}\Theta _{j}\left( \zeta _{k}\right) T_{k}^{*}\left( \zeta \right) \end{aligned}$$

25 $$\begin{aligned} \Phi _{j}\left( \zeta \right) \approx \sum _{k=0}^{P}\Phi _{j}\left( \zeta _{k}\right) T_{k}^{*}\left( \zeta \right). \end{aligned}$$

The *k*th Chebyshev polynomial is defined as

$$\begin{aligned} T_{k}^{*}\left( \zeta \right) =cos\left[ kcos^{-1}\left( \zeta \right) \right]. \end{aligned}$$

The $j{\text {th}}$ derivative of unknown functions $F_{r+1},\; G_{r+1},\;\Theta _{r+1}\; \text {and}\; \Phi _{r+1}$ are constructed as

26 $$\begin{aligned} \left. \begin{array}{ll} \dfrac{d^{j}F_{r+1}}{d\eta ^{j}}=\sum _{k=0}^{P}S_{ki}^{j}f_{r+1}\left( \zeta _k\right) =S^jF_{r+1},\\ \dfrac{d^{j}G_{r+1}}{d\eta ^{j}}=\sum _{k=0}^{P}S_{ki}^{j}g_{r+1}\left( \zeta _k\right) =S^jG_{r+1},\\ \dfrac{d^{j}\Theta _{r+1}}{d\eta ^{j}}=\sum _{k=0}^{P}S_{ki}^{j}\theta _{r+1}\left( \zeta _k\right) =S^j\Theta _{r+1},\\ \dfrac{d^{j}\Phi _{r+1}}{d\eta ^{j}}=\sum _{k=0}^{P}S_{ki}^{j}\phi _{r+1}\left( \zeta _k\right) =S^j\Phi _{r+1}, \end{array}\right\}&\; i=0,1,2\dots P. \end{aligned}$$

Here $S=\dfrac{2D}{L},\quad D$ is Chebyshev differentiation matrix. The entries of this matrix are defined as follows

27 $$\begin{aligned} \left. \begin{array}{ll} D_{00}=\dfrac{2P^{2}+1}{6},\quad D_{ik}=\dfrac{c_i(-1)^{i+k}}{c_k(\zeta _i-\zeta _k)},\qquad i\ne k;i,k=0,1\dots P\\ D_{PP}=-\dfrac{2P^{2}+1}{6},\quad D_{kk}=-\dfrac{\zeta _k}{2(1-\zeta _k^2)}, \qquad k=1,2\dots P-1 \end{array}\right\} \end{aligned}$$

$$\begin{aligned} c_i = {\left\{ \begin{array}{ll} 2 &{}\quad i=0\; \text { or}\; P\\ 1 &{}\quad \text{ otherwise }\\ \end{array}\right. } \end{aligned}$$

In this procedure, we obtain following matrix equation:

28 $$\begin{aligned} \begin{bmatrix} A_{11} &{}\quad A_{12} &{}\quad A_{13} &{}\quad A_{14}\\ A_{21} &{}\quad A_{22} &{}\quad A_{23} &{}\quad A_{24}\\ A_{31} &{}\quad A_{32} &{}\quad A_{33} &{}\quad A_{34}\\ A_{41} &{}\quad A_{42} &{}\quad A_{43} &{}\quad A_{44} \end{bmatrix} \begin{bmatrix} F_{r+1}\\ G_{r+1}\\ \Theta _{r+1}\\ \Phi _{r+1}\\ \end{bmatrix}=\begin{bmatrix} R_{1,r}\\ R_{2,r}\\ R_{3,r}\\ R_{4,r}\\ \end{bmatrix} \end{aligned}$$

where each $A_{ij}$ is of order $(P+1)\times (P+1)$ and the order of each $R_{1,r},\;R_{2,r},\;R_{3,r}\;\text {and}\;R_{4,r}$ is $(P+1)\times 1$.

$$\begin{aligned} \left. \begin{array}{ll} F_{r+1}=\left[ f_{r+1}(\zeta _0),f_{r+1}(\zeta _{1}),\dots f_{r+1}(\zeta _P)\right] ^{T},\\ G_{r+1}=\left[ g_{r+1}(\zeta _0),g_{r+1}(\zeta _{1}),\dots g_{r+1}(\zeta _P)\right] ^{T},\\ \Theta _{r+1}=[\theta _{r+1}(\zeta _0),\theta _{r+1}(\zeta _{1}),\dots \theta _{r+1}(\zeta _P)]^T,\\ \Phi _{r+1}=\left[ \phi _{r+1}(\zeta _0),\phi _{r+1}(\zeta _{1}),\dots \phi _{r+1}(\zeta _P)\right] ^{T},\\ R_{1,r}=[r_{1,r}(\zeta _0),r_{1,r}(\zeta _{1}),\dots ,r_{1,r}(\zeta _P)]^T,\\ R_{2,r}=[r_{2,r}(\zeta _0),r_{2,r}(\zeta _{1}),\dots ,r_{2,r}(\zeta _P)]^T,\\ R_{3,r}=[r_{3,r}(\zeta _0),r_{3,r}(\zeta _{1}),\dots ,r_{3,r}(\zeta _P)]^T,\\ R_{4,r}=[r_{4,r}(\zeta _0),r_{4,r}(\zeta _{1}),\dots ,r_{4,r}(\zeta _P)]^T,\\ A_{11}=a_{113,r}S^3+a_{112,r}S^2+a_{111,r}S+a_{110,r}I,\\ A_{12}=a_{120,r}I,\\ A_{13}=a_{130,r}I,\\ A_{14}=O,\\ A_{21}=a_{211,r}S+a_{210,r}I,\\ A_{22}=a_{222,r}S^{2}+a_{221,r}S+a_{220,r}I,\\ A_{23}=O\\ A_{24}=O,\\ A_{31}=a_{310,r}I+a_{311,r}S+a_{312,r}S^{2}\\ A_{32}=a_{320,r}I+a_{321,r}S,\\ A_{33}=a_{332,r}S^{2}+a_{331,r}S+a_{330}I,\\ A_{34}=a_{341,r}S,\\ A_{41}=a_{410,r}I,\\ A_{42}=O,\\ A_{43}=a_{432,r}S^2\\ A_{44}=S^{2}+a_{441,r}S. \end{array}\right\}. \end{aligned}$$

## Solution error

The solution error method is used to justify the convergence of the solutions for validating our results. In this method, the norm of the difference of the solutions at two consecutive iterations is calculated. If this norm approaches to very small value, then the method converges, and it validates the results obtained by using spectral quasi-linearization method (SQLM). The errors in the solutions of $f(\eta ),\;g(\eta ),\;\theta (\eta )\;\text {and}\;\phi (\eta )$ are defined as

$$\begin{aligned} \text {error}\;F=\Vert f_{r+1}-f_{r}\Vert _{\infty },\;\text {error}\;G=\Vert g_{r+1}-g_{r}\Vert _{\infty },\; \text {error}\;\Theta =\Vert \theta _{r+1}-\theta _{r}\Vert _{\infty }, \text {error}\;\Phi =\Vert \phi _{r+1}-\phi _{r}\Vert _{\infty }. \end{aligned}$$

**Figure 2:**
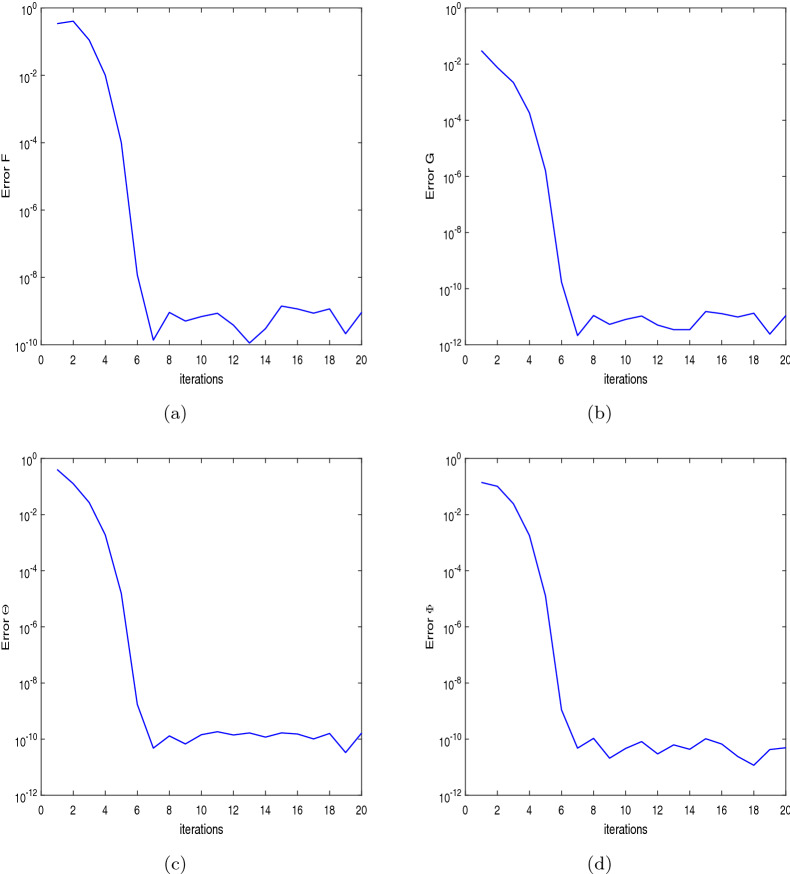
Solution error for (**a**) $f(\eta ),$ (**b**) $g(\eta )$, (**c**) $\theta (\eta ),$ (**d**) $\phi (\eta )$.

The errors in the solutions are represented through the Fig. 2a–d. From these figures, it is concluded that after seven iterations, the error of each solution attains to become less than $10^{-8}$, which validates our results.

## Validation of approximate solutions

In order to validate the present results, a comparison of the present results with the results obtained by Khan and Pop^3^ is executed after neglecting certain parameters. Table 3 represents the comparison of the present results of local Nusselt number and Sherwood number against the pertinent parameter *Nt* at $\beta \rightarrow \infty ,\;Pr=10,\;Sc=10,\;Nb=0.1$ with Khan and Pop by nullifying extra parameters. Moreover, the comparison of the magnitude of skin friction coefficient in *x*-direction against the significant parameters $M \;\text {and}\; \beta$ with the results of Nadeem et al.^34^ is fulfilled through Table 4 by vanishing other parameters. In both comparisons, an appropriate resemblance is observed, which validate the present results.

## Analysis of entropy generation

The study on the entropy generation of any system is prominent to explain the irreversibility of thermal energy in the system. The main objective of the present model is to minimize entropy generation for obtaining better outcomes by controlling several physical parameters. The entropy generation rate per unit volume of the present model can be constructed mathematically as follows:

29 $$\begin{aligned} E_{G}&=\frac{1}{T_{\infty }^{2}}\left( \kappa _{nf}+\dfrac{16\sigma ^*T^3}{3\alpha ^*}\right) \left( \frac{\partial {T}}{\partial {y}}\right) ^2+\frac{\mu _{nf}}{T_{\infty }}\left( 1+\frac{1}{\beta }\right) \left[ \left( \frac{\partial {u}}{\partial {y}}\right) ^2+\left( \frac{\partial {w}}{\partial {y}}\right) ^2\right] \nonumber \\ &\quad +\frac{\sigma B^2(t)}{(1+m^2)T_{\infty }}\left( u^2+w^2\right) +\frac{RD_{B}}{C_{\infty }}\left( \frac{\partial {C}}{\partial {y}}\right) ^2+\frac{RD_{B}}{T_{\infty }}\left( \frac{\partial {T}}{\partial {y}}\right) \left( \frac{\partial {C}}{\partial {y}}\right) \end{aligned}$$

- The term $\frac{1}{T_{\infty }^{2}}\left( \kappa _{nf}+\dfrac{16\sigma ^*T^3}{3\alpha ^*}\right) \left( \frac{\partial {T}}{\partial {y}}\right) ^2$ represents entropy generation due to heat transfer irreversibility.
- The term $\frac{\mu _{nf}}{T_{\infty }}\left( 1+\frac{1}{\beta }\right) \left[ \left( \frac{\partial {u}}{\partial {y}}\right) ^2+\left( \frac{\partial {w}}{\partial {y}}\right) ^2\right]$ presents entropy generation due to the viscous dissipation of energy of the Casson nanofluid.
- The term $\frac{\sigma B^2(t)}{(1+m^2)T_{\infty }}\left( u^2+w^2\right)$ signifies entropy generation due to the applied magnetic field.
- The terms $\frac{RD_{B}}{C_{\infty }}\left( \frac{\partial {C}}{\partial {y}}\right) ^2$ and $\frac{RD_{B}}{T_{\infty }}\left( \frac{\partial {T}}{\partial {y}}\right) \left( \frac{\partial {C}}{\partial {y}}\right)$ indicate the entropy generation because of the mass transfer irreversibility.

The non dimensional form of entropy generation can be expressed as follows:

30 $$\begin{aligned} N_{G}=\frac{E_{G}}{E_{G0}}=\dfrac{T^2_{\infty }\left( \frac{y}{ \eta }\right) ^2}{\kappa _{nf}\left( T_{w}-T_{\infty }\right) ^2}E_{G} \end{aligned}$$

where $$E_{G0}=\dfrac{\kappa _{nf}\left( T_{w}-T_{\infty }\right) ^2}{T^2_{\infty }\left( \frac{y}{\eta }\right) ^2}.$$

Utilizing the similarity transformation, the nondimensional entropy generation can be reduced to the following form

31 $$\begin{aligned} N_{G}= & {} \underbrace{\left[ 1+\frac{4Nr}{3}\left\{ \theta (tr-1)+1\right\} ^3\right] \theta ^{'2} }_{N_{GT}}\nonumber \\&+\,\underbrace{\left[ \dfrac{MBr}{\left( 1+m^2\right) \alpha _{1}}\left( f^{'2}+g^2\right) +\left( 1+\frac{1}{\beta }\right) \frac{Br}{\alpha _{1}}\left( f^{''2}+g^{'2}\right) \right] }_{N_{GFF}}+\underbrace{\left[ \dfrac{\alpha _{2}^2L}{\alpha _{1}^2}\phi ^{'2}+\dfrac{L\alpha _{2}}{\alpha _{1}}\phi ^{'}\theta ^{'}\right] }_{N_{GM}} \end{aligned}$$

where Brinkman number $Br=\frac{\mu _{nf} u_{w}^2}{\kappa _{nf}\Delta T}=\frac{\mu _{nf} a^2x^2}{\left( 1-\gamma t\right) ^{2}\kappa _{nf}\left( T_{w}-T_{\infty }\right) },$ dimensionless temperature ratio variable $\alpha _{1}=\frac{T_{w}-T_{\infty }}{T_{\infty }},$ dimensionless concentration ratio variable $\alpha _{2}=\frac{C_{w}-C_{\infty }}{C_{\infty }},$ diffusive variable $L=\frac{RD_{B}C_{\infty }}{\kappa _{nf}}.$ $N_{G}$ is total entropy generation of the system. $N_{GT}$ is entropy number due to thermal irreversibility. $N_{GFF}$ defines entropy generation number due to fluid friction irreversibility including the impact of applied magnetic field. $N_{GM}$ denotes entropy number due to mass transfer irreversibility. A significant parameter in the analysis of entropy generation is Bejan number. Taking care of it, the dimensionless Bejan number is constructed mathematically as follows:

32 $$\begin{aligned} Be=\frac{N_{GT}}{N_{G}}=\frac{N_{GT}}{N_{GT}+N_{GFF}+N_{GM}}=\frac{\text {Entropy generation due to heat transfer}}{\text {Total entropy generation}} \end{aligned}$$

From Eq. (32), it is clear that Bejan number ranges from 0 to 1. If $Be\ll 0.5$, entropy generation due to friction irreversibility dominates over heat transfer irreversibility. If $Be\gg 0.5$, then entropy generation due to heat transfer irreversibility dominates over friction irreversibility. For $Be=0.5$, the heat transfer irreversibility and friction irreversibility are equal.

## Results and discussion

The numerical study of the present mathematical model is analyzed by taking the effects of Hall current, radiation, mixed convection, heat generation, viscous and Joule dissipations, Brownian motion and thermophoresis into account under some boundary conditions. The present model of the physical problem is characterized by a set of time and space dependent nonlinear partial differential equations containing momentum equation, energy equation and concentration equation. Similarity transformations are applied to obtain a set of nonlinear ordinary differential equations, and SQLM is used to solve these ordinary differential equations subject to the relevant boundary conditions. From the physical point of view, the impact of several values of specified parameters on the flow field, such as mixed convection parameter, Prandtl number, magnetic number, Eckert number, Brownian motion parameter, thermophoretic parameter, Schmidt number, Hall current parameter, Radiation parameter and heat generation parameter are explored and plotted graphically. In the current section, for the numerical computation, the default values of pertinent parameters are taken as $A=0.1,\; M=7,\; m=0.2,\;\beta = 0.3,\; Nr=0.5,\; Pr=6,\; tr=1,\; Sc=1.5,\; Nb=0.2,\; Nt=0.1,\; \lambda =8,\; Ec=0.1\;\text {and} \; \alpha =0.1.$

### Nanofluid velocity profile

**Figure 3:**
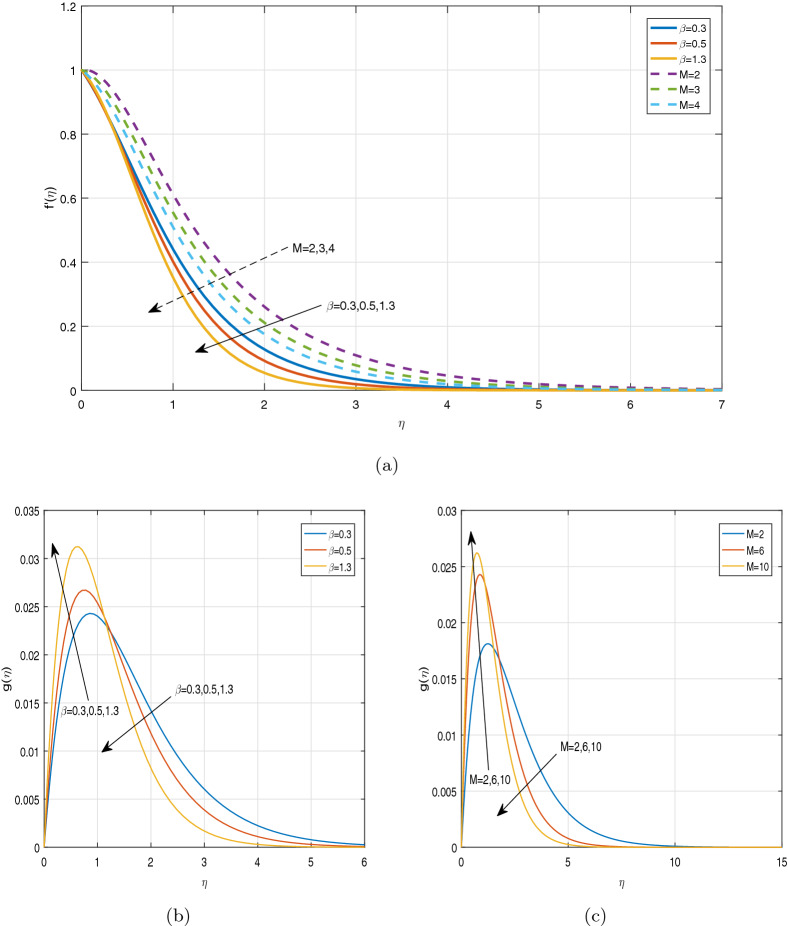
Graphs of (**a**) $f^{'}(\eta )$ against $\beta$ and *M*, (**b**) $g(\eta )$ against $\beta$, (**c**) $g(\eta )$ against *M*.

**Figure 4:**
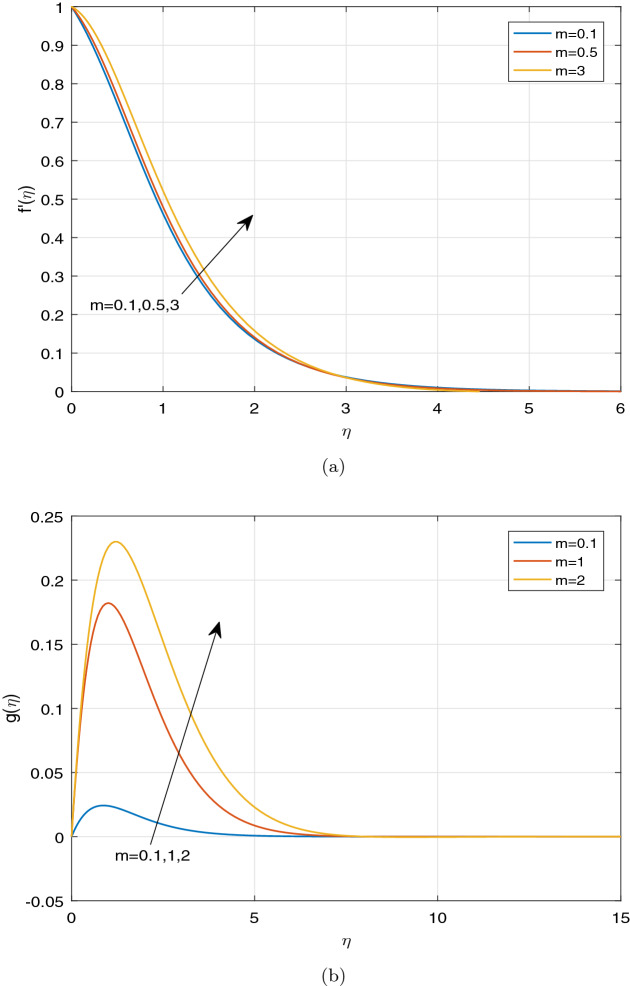
Impact of *m* on (**a**) $f^{'}(\eta )$, (**b**) $g(\eta )$.

**Figure 5:**
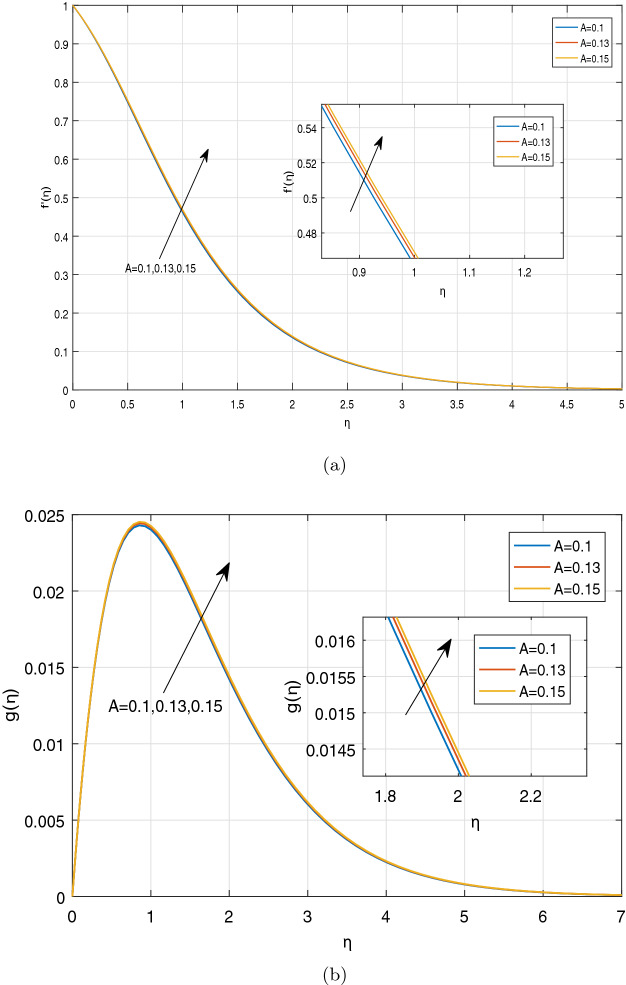
Impact of A on (**a**) $f^{'}(\eta )$, (**b**) $g(\eta )$.

**Figure 6:**
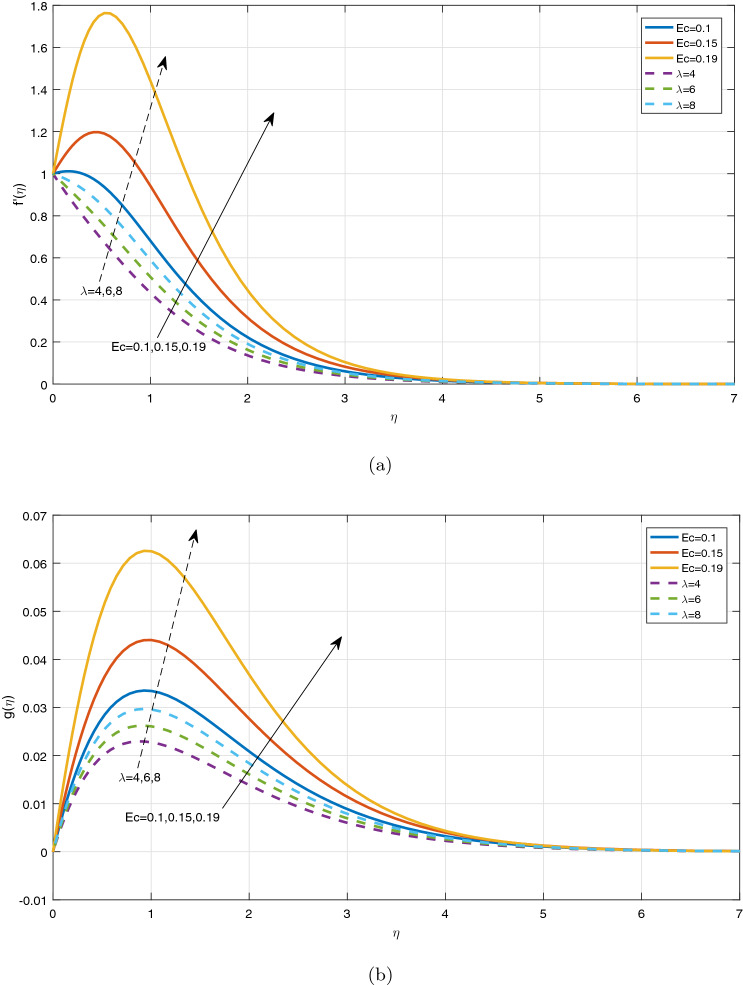
Impact of *Ec* and $\lambda$ on (**a**) $f^{'}(\eta )$, (**b**) $g(\eta )$.

Figures 3, 4, 5 and 6 depict the impact of disparate values of pertinent parameters on the nanofluid velocity profiles. The impact of Casson parameter $\beta$ and magnetic parameter *M* on the profiles of velocity components in x and z-directions is depicted graphically in Fig. 3a–c, respectively. It is visualized from these figures that raising the values of both Casson parameter $\beta$ and magnetic field parameter *M* leads to reduce the velocity $f^{'}(\eta )$ in *x*-direction within the boundary layer region. When the Casson parameter $\beta$ tends to $\infty$, the fluid turns into a Newtonian fluid. The increase in the values of $\beta$ enhances the plastic dynamic viscosity, and hereby the yield stress diminishes. This resists the fluid motion. The presence of magnetic field in an electrically conducting fluid creates a resistive force called Lorentz force. This force retards the motion of nanofluid, and as a result, the velocity $f^{'}(\eta )$ in *x*-direction gets decreased with increasing the values of magnetic parameter *M*. On the contrary, the dual nature of transverse velocity ($g(\eta )$) can be observed for increasing the values of magnetic parameter and $\beta$. On increasing the magnetic parameter and $\beta$,the nanofluid velocity $g(\eta )$ in *z*-direction gets increased rapidly near the sheet, and after that, it dwindles away from the sheet within the boundary layer. The strong magnetic field *B*(*t*) applied on the flow of an electrically conducting nanofluid produces Hall current and the impact of this current on the profiles of nanofluid velocity is demonstrated graphically in the Fig. 4a, b. It is witnessed that the Hall current parameter has no considerable effect on the nanofluid velocity $f^{'}(\eta )$ in *x*-direction. However, the small increment in $f^{'}(\eta )$ with increasing the values of Hall current parameter *m* can be visualized. The nanofluid velocity $g(\eta )$ in *z* direction enhances considerably on increasing the Hall current parameter *m*. Physically, lessening the conductivity $(\frac{\sigma }{1+m^2})$ means strengthening the Hall current parameter *m*, which generates a magnetic damping force caused to speed up the velocity components of the nanofluid. The impact of unsteadiness parameter A on the velocity profiles can be observed in Fig. 5a, b. It is evident that on increasing unsteadiness parameter A, the velocity components are increasing slowly, which leads to rise the thickness of momentum boundary layer. Figure 6a, b display the velocity distribution for various values of Eckert number and mixed convection parameter $\lambda$. It is concluded from the figures that the increment in parameter Ec means strengthening the kinetic energy, which enhances the velocity components in *x* and *z* directions. It is also evident that the increment in Ec increases the boundarylayer thickness. It is observed from Fig. 6a, b that mixed convection parameter $\lambda$ has a tendency to enhance the fluid velocity. This phenomenon happens only in the presence of Buoyancy force.

### Nanofluid temperature profile

**Figure 7:**
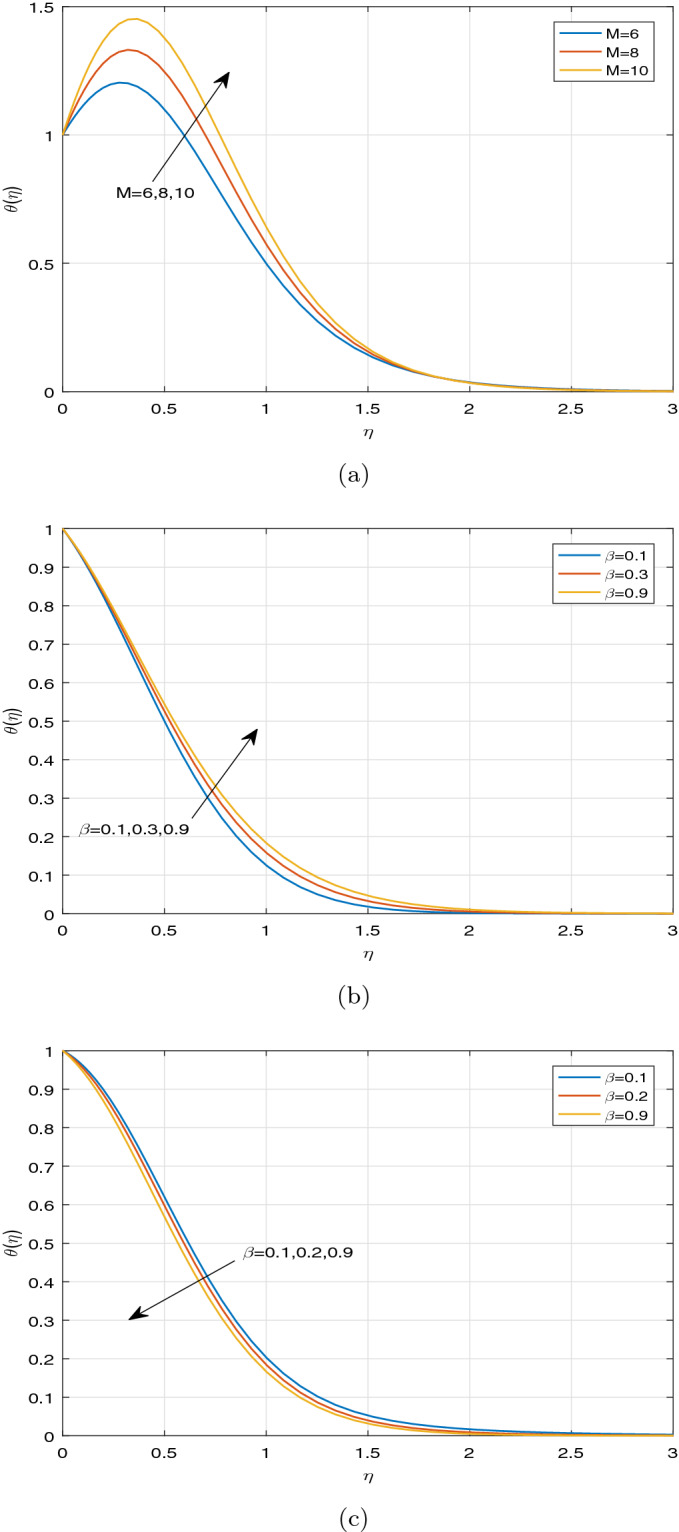
Temperature profiles against (**a**) M, (**b**) $\beta \;\text {and}\; Ec=0$, (**c**) $\beta \;\text {and}\; Ec>0$.

**Figure 8:**
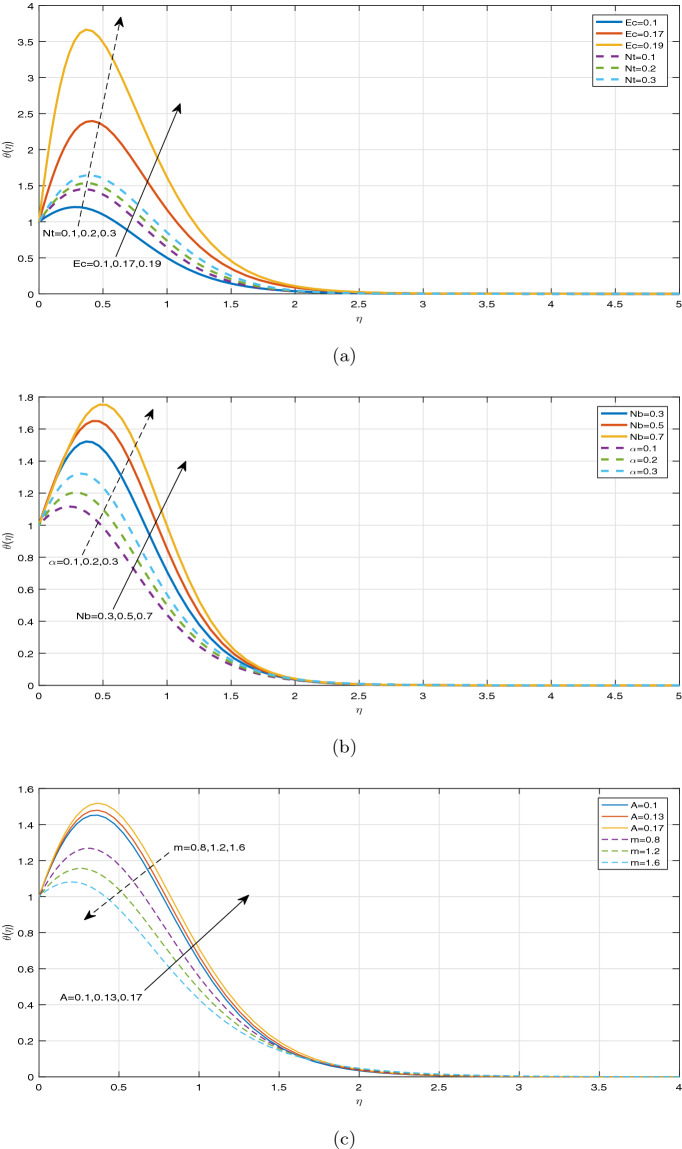
Temperature profiles against (**a**) *Ec* and *Nt*, (**b**) *Nb* and $\alpha$, (**c**) *A* and *m*.

**Figure 9:**
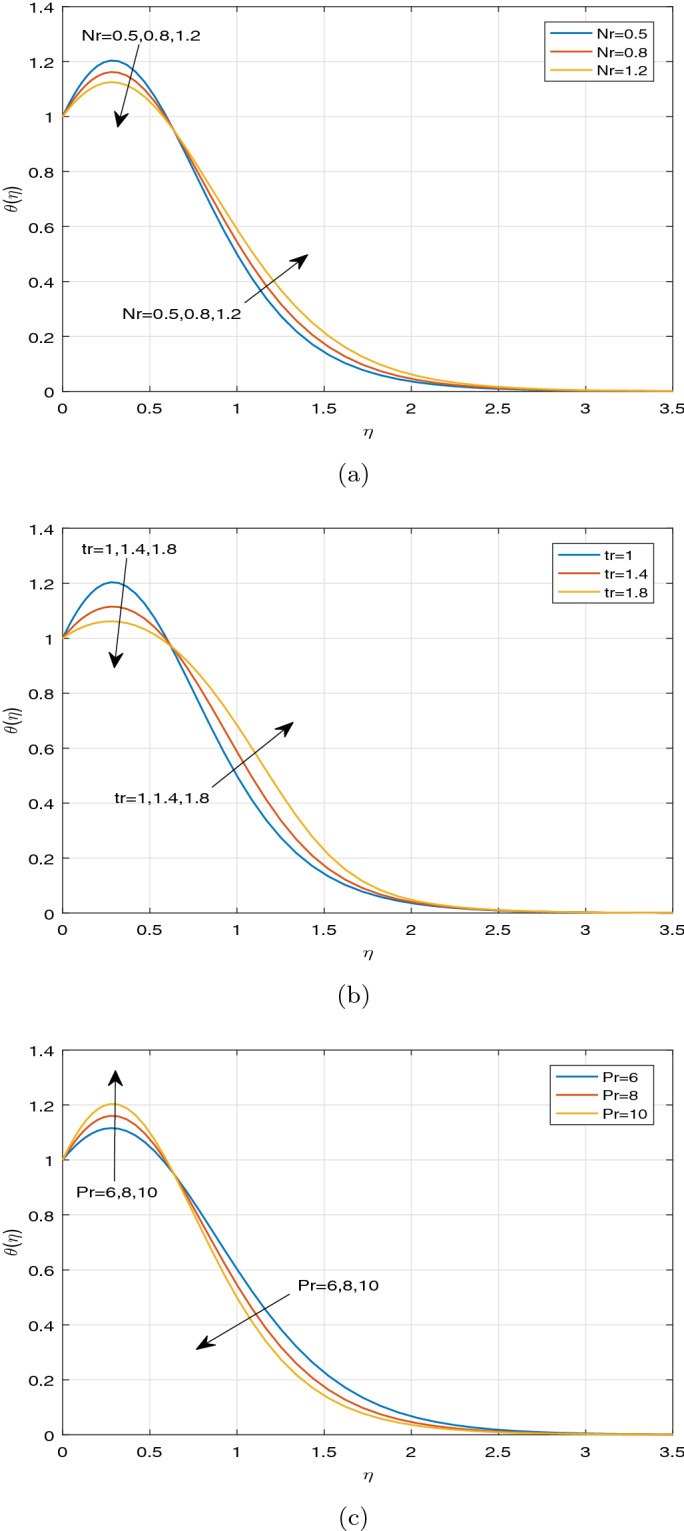
Temperature profiles against (**a**) *Nr*, (**b**) tr, (**c**) Pr.

Figures 7, 8 and 9 display the influence of some specified parameters on Casson nanofluid temperature profiles within the thermal boundary layer. It is observed from Fig. 7a that magnetic parameter M has a tendency to enhance the nanofluid temperature within the boundary layer. The applied magnetic field boosts to increase Ohmic heating, which is produced by Lorentz force. As a result, temperature rises on increasing magnetic parameter. The temperature profiles for different values of Casson parameter $\beta$ in the cases, such as $Ec=0$ and $Ec> 0$ are depicted in Fig. 7b, c, respectively. It is evident that the increment in Casson parameter $\beta$ leads to increase the nanofluid temperature in the absence of Ec and diminishes the nanofluid temperature in the presence of Ec within the boundary layer. Since Casson parameter $\beta$ reduces the velocity, and Eckert number is inversely proportional to the temperature difference, it resembles that Casson parameter has a tendency to diminish the temperature under the impact of viscous and Joule dissipations $(Ec>0)$. Figure 8a portrays the impact of various values of Ec and thermophoretic parameter Nt on nanofluid temperature profiles. It can be visualized that increasing the values of Ec leads to enhance temperature profile throughout the boundary layer. On increasing Ec, a hump is noted in the region near the sheet, and after that, nanofluid temperature tends to ambient temperature value away from the sheet. Eckert number is the ratio of the kinetic energy to the enthalpy. However, the impact of Ec on the temperature profile is noticed only due to the increasing trend of viscous and Joule dissipations. In case of viscous and Ohmic heating, as a consequence of dissipation effects, heat is generated due to friction between two adjacent electrically conducting fluid layers and hereby fluid temperature rises. It can be seen that the parameter Nt has an increasing effect on temperature distribution. As a result, the thermal boundary layer thickness improves significantly. Physically, the increment in Nt occurs by means of the enhancement in the thermophoretic phenomenon. Thermophoresis is one type of particle motion under the influence of the applied thermal gradients, whereas it is related deeply to the soret effect. Nanoparticles transfer thermal energy from the hotter side to the cooler side within the boundary layer region due to diffusion of particles caused by the thermophoretic effect. Thus the temperature of fluid increases significantly. Figure 8b illustrates the temperature profile for disparate values of Nb. It is evident from Fig. 8b that the increment in Nb leads to increase fluid temperature. This phenomenon represents an increase of Brownian motion, which indicates the irregular movement of particles suspended in the fluid. It can be concluded that increasing the Brownian motion enhances temperature considerably throughout the boundary layer due to increasing the collision between fluid particles. Also Fig. 8b exhibits the heat generation effect on the temperature profile in the presence of Buoyancy force. It is observed that ascending the values of heat generation parameter enhances fluid temperature significantly. It is due to the fact that the external heat source introduces more heat in the flow region, as a result of which, the fluid temperature increases. Figure 8c predicts the impact of the temperature distribution against the unsteadiness parameter A and the Hall current parameter m. It is noticeable that the unsteadiness parameter A has no significant effect on the fluid temperature. A minor increment of temperature is visualized for increasing the values of the unsteadiness parameter A throughout the boundary layer. As a result, the thickness of thermal boundary layer enhances. Meanwhile, it is depicted from Fig. 8c that the Hall current parameter m has a strictly decreasing affect on the distribution of temperature throughout the boundary layer. Moreover, the impact of radiative parameter Nr, the temperature ratio parameter *tr* and Prandtl number Pr on the fluid temperature is portrayed in Fig. 9a–c, respectively. From Fig. 9a, the decreasing behaviour of temperature distribution towards the radiative parameter Nr can be noticed near the sheet. But away from the sheet, temperature is highly rising on increasing the radiative parameter Nr. At a certain distance from the sheet, the fluid absorbs more heat due to a larger radiation parameter. This fact is responsible to increase temperature far from the sheet. From the Fig. 9b, the similar observation can be visualized in case of temperature distribution against the parameter *tr*. The temperature ratio parameter *tr* indicates the ratio of the fluid temperature at the surface to the fluid temperature outside the boundary layer region. From Fig. 9c, it can be noticed the opposite observation on temperature profile against Prandtl number. Prandtl number indicates the ratio of momentum diffusivity to thermal diffusivity. The increment in Prandtl number signifies that the higher momentum diffusivity drops fluid temperature. This fact clarifies that fluid temperature is strictly rising under the increasing effect of thermal diffusivity throughout the boundary layer region.

### Nanofluid concentration profile

**Figure 10:**
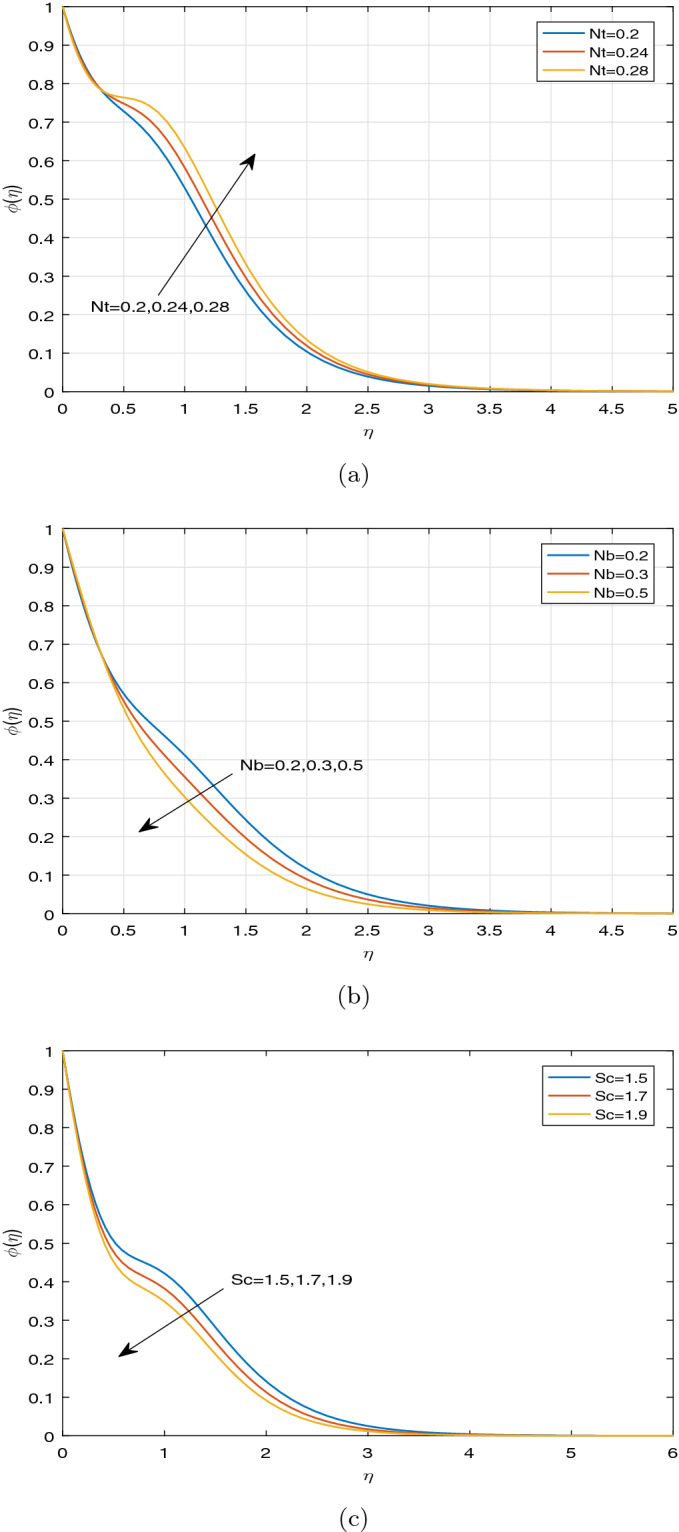
Concentration profiles against the disperate values of pertinent parameters (**a**) Nt, (**b**) Nb, (**c**) Sc.

Figure 10a–c display the influence of some specified parameters on Casson nanofluid concentration profiles throughout the boundary layer. Figure 10a depicts the nanoparticle concentration profile for different values of *Nt*. It is ascertained that *Nt* acts as an assisting parameter in case of concentration distribution inside the boundary layer region. From the physical point of view, this observation occurs due to the increment of the thermophoretic phenomena. Moreover, Fig. 10b illustrates to highlight the nanoparticle concentration profile for various values of Nb. Physically, the enhancement in parameter Nb refers to occur the collision between nanoparticles repeatedly. As a result of which, the species between nanoparticles diminishes, which leads to decrease the concentration distribution. From Fig. 10c, it is demonstrated that the larger Schmidt number *Sc* is responsible for decreasing the concentration within the boundary layer, which results in thinning the thickness of nanoparticle concentration boundary layer. The larger Schimdt number means the lesser mass diffusivity, which indicates to decrease the nanoparticle concentration throughout the boundary layer.

### Entropy generation and Bejan number

**Figure 11:**
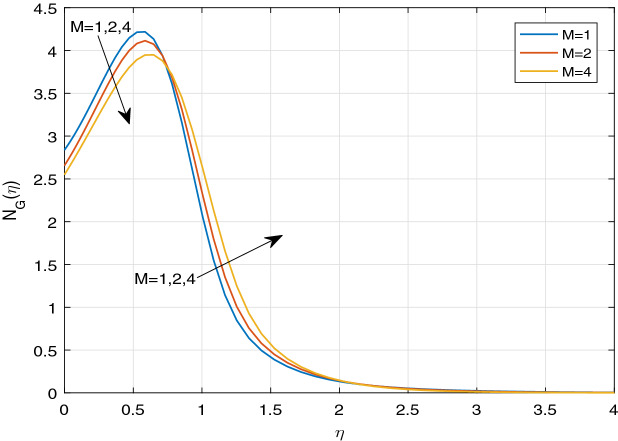
Sketch of $N_{G}$ against *M*.

**Figure 12:**
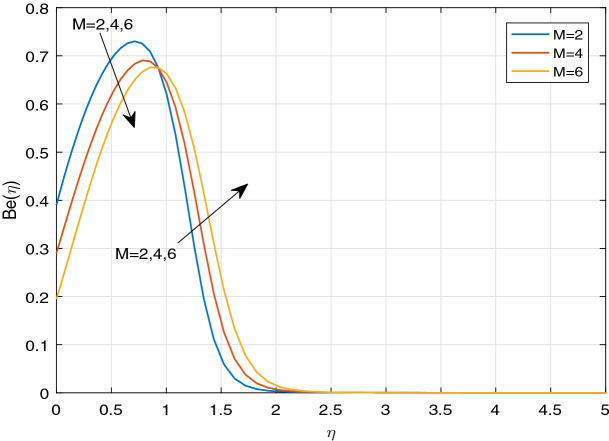
Sketch of *Be* against *M*.

**Figure 13:**
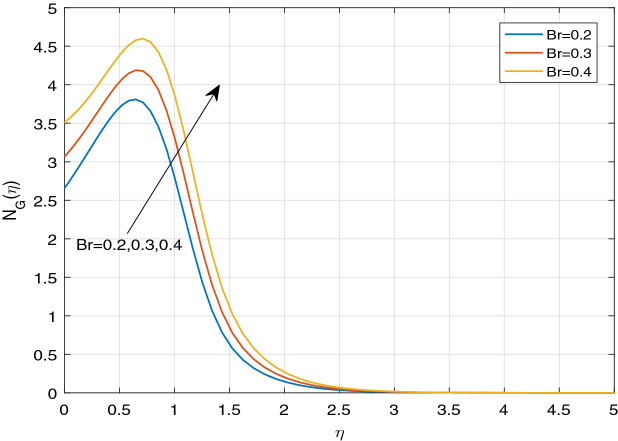
Sketch of $N_{G}$ against *Br*.

**Figure 14:**
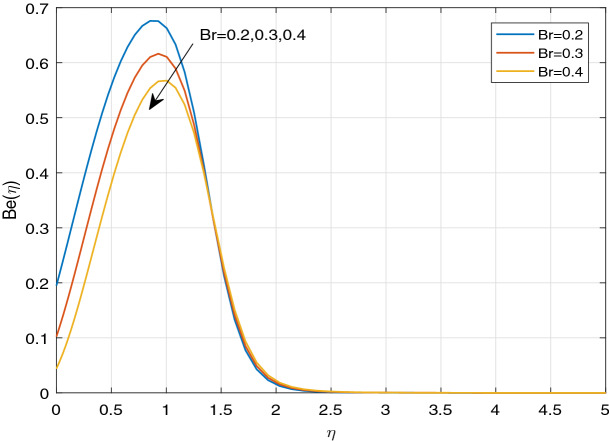
Sketch of *Be* against *Br*.

**Figure 15:**
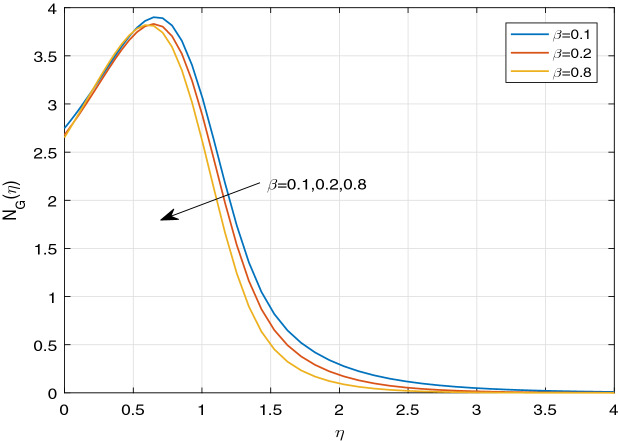
Sketch of $N_{G}$ against $\beta$.

**Figure 16:**
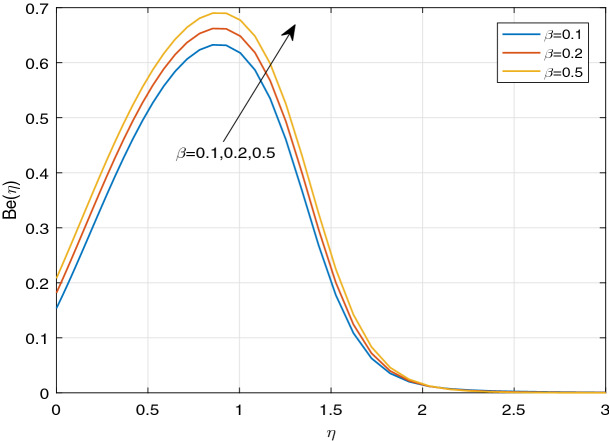
Sketch of *Be* against $\beta$.

**Figure 17:**
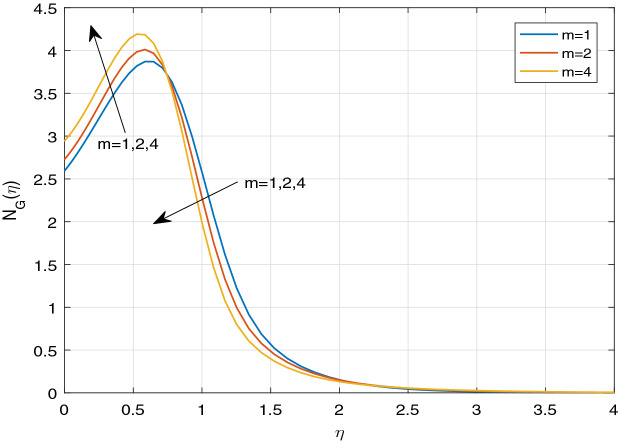
Sketch of $N_{G}$ against *m*.

**Figure 18:**
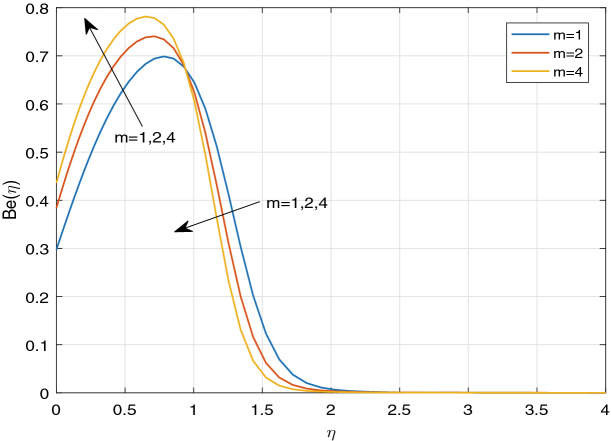
Sketch of *Be* against *m*.

**Figure 19:**
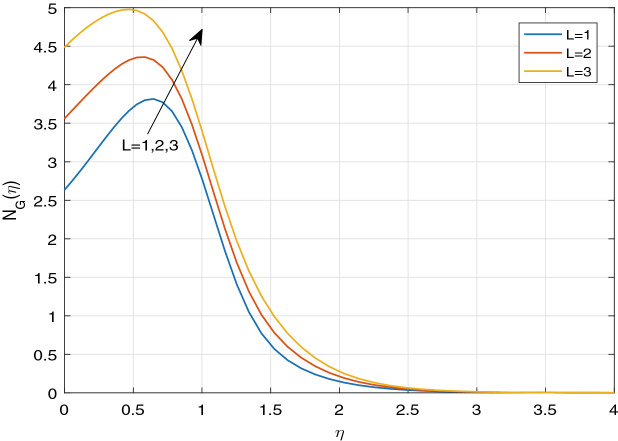
Sketch of $N_{G}$ against *L*.

**Figure 20:**
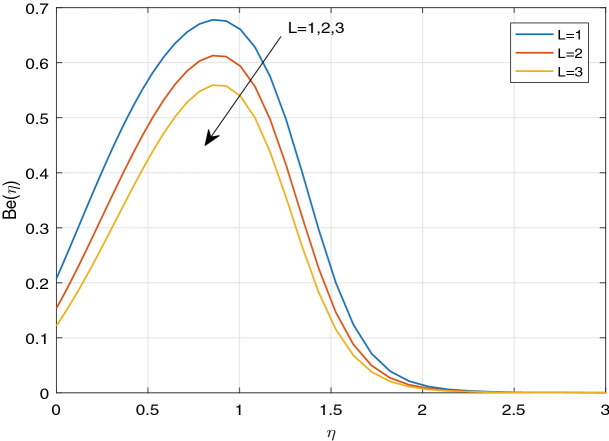
Sketch of *Be* against *L*.

**Figure 21:**
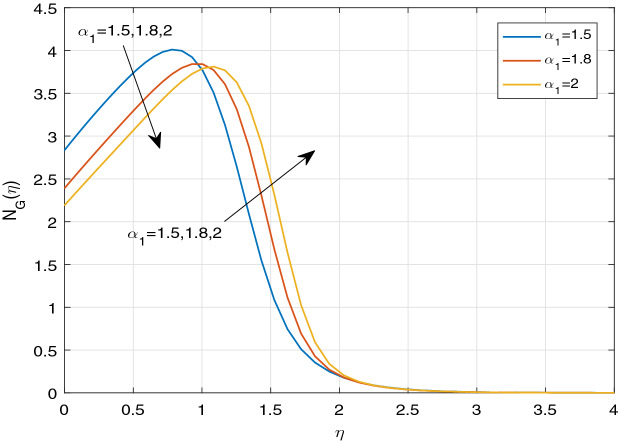
Sketch of $N_{G}$ against $\alpha _{1}$.

**Figure 22:**
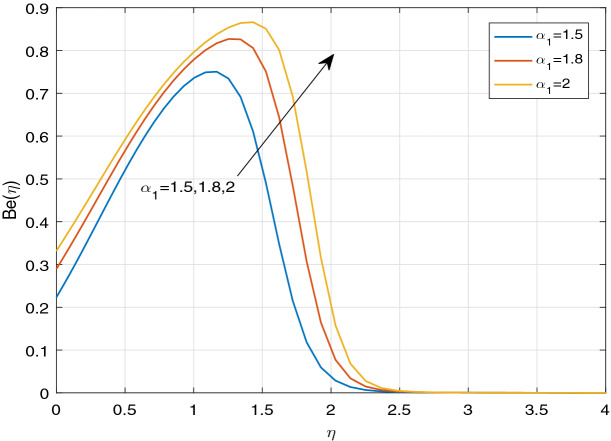
Sketch of *Be* against $\alpha _{1}$.

**Figure 23:**
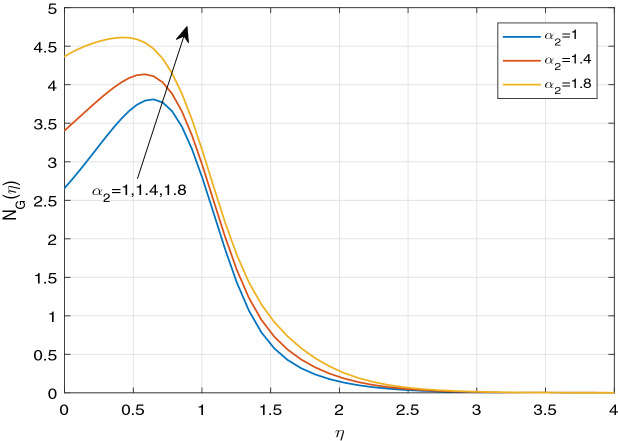
Sketch of $N_{G}$ against $\alpha _{2}$.

**Figure 24:**
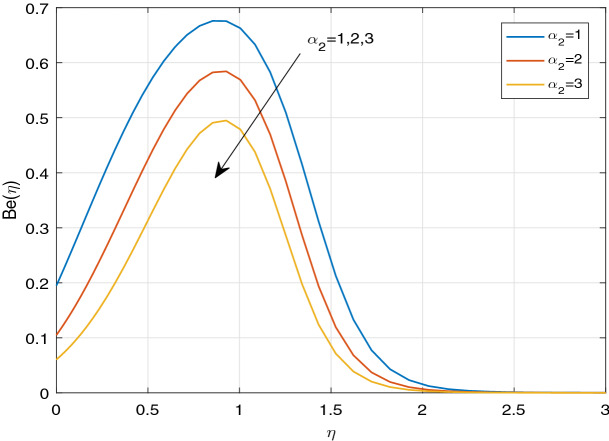
Sketch of *Be* against $\alpha _{2}$.

**Figure 25:**
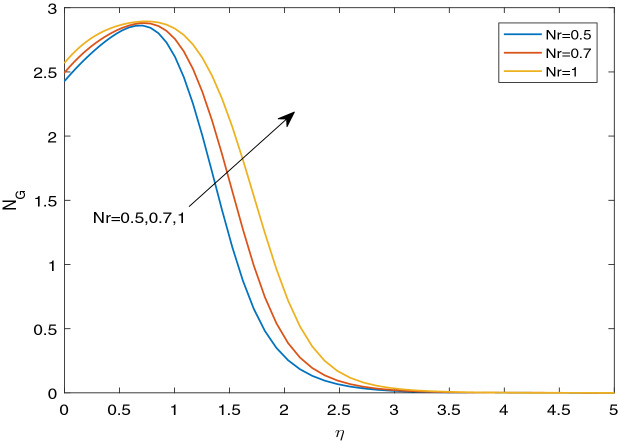
Sketch of $N_{G}$ against *Nr*.

**Figure 26:**
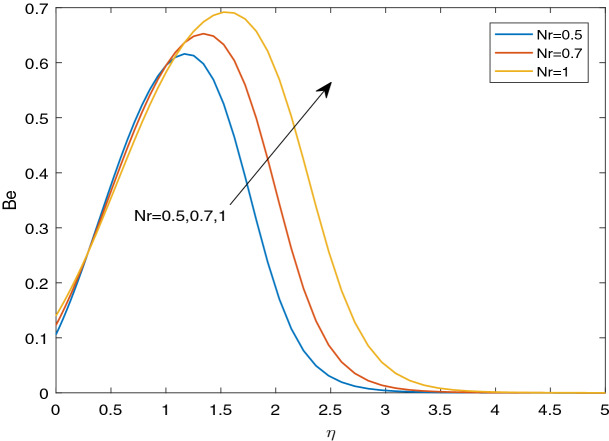
Sketch of *Be* against *Nr*.

Figures 11, 12, 13, 14, 15, 16, 17, 18, 19, 20, 21, 22, 23, 24, 25 and 26 illustrate the influences of pertinent parameters on the entropy generation. Figures 11 and 12 are shown to exhibit the impact of magnetic parameter *M* on entropy generation $N_{G}$ and Bejan number *Be*. It is noticed from Fig. 11 that initially the increment in magnetic parameter decreases the entropy generation near the sheet, and then leads to enhance the entropy generation. From Fig. 12, in case of Bejan number, the same fact is visualized. For the large values of M, the resistive Lorentz force is generated, which retards the fluid motion, and in the presence of a strong applied magnetic field, the temperature increases due to Ohmic heating, which leads to introduce much heat. Hereby entropy generation increases. Away from the sheet, for the large M, heat transfer irreversibility dominates over fluid friction irreversibility. As a result, Bejan number increases. Figure 13 elaborates the variation of entropy profile against the multiple values of Brinkman number. Brinkman number is the ratio of heat produced by viscous dissipation to heat transported by molecular conduction. Physically, on increasing the Brinkman number, the conduction rate of the heat generated due to viscous dissipation depreciates, resulting in an enhancement in the entropy generation rate. The behaviour of Bejan number for multiple values of Brinkman number is plotted in Fig. 14. Here the graph of Bejan number declines for increasing the Brinkman number. Physically, the total entropy generation rate rises due to the increment in Brinkman number, which leads to decline the Bejan number. Figure 15 demonstrates the impact of various values of Casson parameter $\beta$ on entropy generation profile. It is analyzed that on increasing the values of Casson parameter $\beta$, entropy generation rate decays. Physically, this phenomenon occurs due to the stress of Casson liquid, which reduces rheological features. When Casson parameter approaches $\infty$, the fluid behaves as a Newtonian fluid. As a result, the liquid has a tendency to shear quickly along the sheet, which causes to decrease entropy generation rate. The impact of Casson parameter on Bejan number is exposed through the Fig. 16. On increasing the Casson parameter, the increasing trend of Bejan number is visualized. When Casson parameter becomes large, then the total entropy generation rate decays. Therefore Bejan number rises. Figure 17 depicts the impact of various values of Hall current parameter *m* on entropy generation profile. The increment in Hall current parameter enhances the entropy generation near the sheet, but at a certain distance from the sheet, the entropy generation diminishes significantly. In case of Bejan number, the same phenomenon is observed through the Fig. 18. At the neighbourhood of the sheet, the entropy generation due to thermal irreversibility dominates over fluid friction irreversibility for increasing the values of Hall current parameter. So Bejan number increase near the sheet. Far from the sheet, increasing the values of Hall current parameter decays temperature. As a result of which, the entropy generation decreases. Also the entropy generation due to thermal irreversibility is dominated by fluid friction irreversibility on increasing *m* away from the sheet. As a result, Bejan number decreases. Figures 19 and 20 depict the effect of diffusive variable *L* on $N_{G}$ and *Be*, respectively. Figure 19 shows that $N_{G}$ gets increased with increasing the values of *L*. Figure 20 depicts that the enhancement in *L* decreases *Be*. For the large *L*, the mass diffusivity of nanoparticle increases, which causes to rise the entropy generation. From the physical point of view, since the total entropy generation rate enhances, Bejan number decays. Figures 21 and 22 are drawn to highlight the behavior of $N_{G}$ and *Be* against the distinct values of temperature ratio parameter $\alpha _{1}$. It is worth noting that on increasing the values of $\alpha _{1}$, the entropy generation gets decreased near the sheet, however, later it increases significantly away from the sheet. From Fig. 22, it can be seen that Bejan number has a significantly increasing effect on the enhancement in the temperature ratio parameter $\alpha _{1}$. For the large values of $\alpha _{1}$, the thermal irreversibility dominates over the fluid friction irreversibility. Therefore Bejan number gets increased. The Influence of concentration ratio parameter $\alpha _{2}$ on $N_{G}$ and *Be* is elaborated through the Figs. 23 and 24, respectively. It is evident that $N_{G}$ gets enhanced for increasing the values of concentration ratio parameter $\alpha _{2}$ by means of increasing the mass transfer irreversibility. However, Fig. 24 depicts that the increment in concentration ratio parameter $\alpha _{2}$ reduces the Bejan number. Physically, since the total entropy generation rate increases, the Bejan number decays. From Figs. 25 and 26, it is evident that higher thermal radiation parameter *Nr* has a tendency to enhance the entropy generation and Bejan number rapidly. For the higher estimation of *Nr*, the temperature increases, which results in an increment in entropy generation and Bejan number. Here the thermal irreversibility dominates over the total entropy generation.

### Skin friction, Nusselt number and Sherwood number

For the engineering interest, the effect of disparate values of pertinent parameters on the numerical values of some physical quantities such as skin friction coefficients ($C_fx$ and $C_fz$), local Nusselt number and Sherwood number are displayed in Tables 1 and 2. From Tables 1 and 2 it is visualized that the magnitude of skin friction coefficient in *x* direction is a decreasing function of unsteadiness parameter *A*, Hall current parameter *m*, $\beta , Nr, tr, Ec, \lambda , \alpha , Nt, Nb\;\text {and}\; Sc$. Whereas, the magnitude of skin friction coefficient in *z* direction has a decreasing effect on Casson parameter $\beta$ and Prandtl number *Pr*. Moreover, the favourable behaviour of the parameters $M,\;\text {and}\; Pr$ to the magnitude of skin friction coefficient in *x* direction can be noticed. The parameters $A, M, m,\; Nr,\; tr, \; Ec,\;\lambda ,\; \alpha ,\; Nt,\; Nb\; \text {and}\; Sc$ have an increasing trend towards the magnitude of skin friction coefficient in *z* direction. The parameters $A,\;M,\; Ec,\; Pr,\; \lambda ,\; \alpha ,\; Nt,\; Nb\;\text {and}\; Sc$ have a tendency to increase the rate of heat transfer in magnitude and the opposing nature of the parameters $m,\; \beta ,\; Nr,\; tr$ on the rate of heat transfer in magnitude is observed. However, the parameters $A,\;M,\; Ec,\; Pr,\; \lambda ,\; \alpha ,\; Nt\;\text {and}\;Sc$ lead to enhance the rate of mass transfer in magnitude, whereas, the parameters $m,\; \beta ,\; Nr,\; tr,\;\text {and}\; Nb$ have a tendency to retard the rate of mass transfer in magnitude.

**Table 1:** Numerical values of skin friction coefficients $\left( C_{fx}\;\text {and}\;C_{fz}\right) ,$ Nusselt Number $Nu_x,$ and Sherwood number $Sh_x$ for $Pr=6,\;\lambda =5,\;\alpha =0.2,\; Nt=0.1,\;Nb=0.1,\;Sc=1.5$.

*A*	*M*	*m*	$\beta$	*Nr*	*tr*	*Ec*	$-C_{fx}Re_{x}^{\frac{1}{2}}$	$C_{fz}Re_{x}^{\frac{1}{2}}$	$-Nu_{x}Re_{x}^{\frac{-1}{2}}$	$Sh_{x}Re_{x}^{\frac{-1}{2}}$
0.1	6	0.1	0.3	0.5	1	0.1	2.6500	0.3067	1.0328	1.5524
0.13							2.6178	0.3080	1.0848	1.5611
0.15							2.5949	0.3090	1.1209	1.5671
	6						2.6500	0.3067	1.0328	1.5524
	8						3.3787	0.3584	1.6297	1.8245
	10						4.0431	0.4027	2.1844	2.0828
		0.1					2.6500	0.3067	1.0328	1.5524
		0.5					2.2987	1.3345	0.7671	1.4240
		0.8					1.9058	1.7804	0.4661	1.2826
			0.3				2.6500	0.3067	1.0328	1.5524
			0.5				2.0957	0.2635	0.8442	1.4182
			0.8				1.7364	0.2344	0.7217	1.3257
				0.5			2.6500	0.3067	1.0328	1.5524
				0.8			2.6040	0.3111	0.9804	1.4144
				1.2			2.5464	0.3163	0.9160	1.2992
					1		2.6500	0.3067	1.0328	1.5524
					1.4		2.5802	0.3127	0.9301	1.2691
					2		2.4114	0.3255	0.6749	1.0525
						0.1	2.6500	0.3067	1.0328	1.5524
						0.15	2.1339	0.3243	2.2262	2.2010
						0.2	1.5037	0.3457	3.6082	2.9308

**Table 2:** Numerical values of skin friction coefficients $\left( C_{fx}\;\text {and}\;C_{fz}\right) ,$ Nusselt Number $Nu_x,$ and Sherwood number $Sh_x$ for $A=0.1,\;M=6,\;m=0.1,\;\beta =0.3,\;Nr=0.5,\;tr=1,\;Ec=0.1$.

*Pr*	$\lambda$	$\alpha$	*Nt*	*Nb*	*Sc*	$-C_{fx}Re_{x}^{\frac{1}{2}}$	$C_{fz}Re_{x}^{\frac{1}{2}}$	$-Nu_{x}Re_{x}^{\frac{-1}{2}}$	$Sh_{x}Re_{x}^{\frac{-1}{2}}$
6	5	0.2	0.1	0.1	1.5	2.6500	0.3067	1.0328	1.5524
8						2.6979	0.3017	1.4519	1.7927
10						2.7232	0.2984	1.8679	2.0326
	5					2.6500	0.3067	1.0328	1.5524
	8					0.8971	0.3463	1.0400	1.6354
	9					0.2732	0.3605	1.0996	1.6909
		0.2				2.6500	0.3067	1.0328	1.5524
		0.25				2.5413	0.3104	1.2677	1.6803
		0.28				2.4707	0.3129	1.4189	1.7622
			0.1			2.6500	0.3067	1.0328	1.5524
			0.2			2.5606	0.3104	1.1380	2.4204
			0.3			2.4690	0.3143	1.2417	3.3824
				0.1		2.6500	0.3067	1.0328	1.5524
				0.2		2.5547	0.3102	1.1477	1.2028
				0.3		2.4625	0.3136	1.2388	1.0855
					1.5	2.6500	0.3067	1.0328	1.5524
					2	2.6407	0.3070	1.0455	1.7210
					2.5	2.6367	0.3071	1.0506	1.8607

## Quadratic multiple regression analysis

In the present section, the skin friction coefficients $\left( C_{fx}\;\text {and}\;C_{fz}\right)$, the reduced Nusselt number and the reduced Sherwood number are estimated with the help of quadratic multiple regression method. The approximated quadratic regression models for $C_{fx}Re^{1/2}_{x},\;C_{fz}Re_{x}^{1/2},\;Nu_{x}Re^{-1/2}_{x}\;\text {and}\;Sh_{x}Re^{-1/2}_{x}$ are constructed, respectively, as follows:

33 $$\begin{aligned} C_{fx_{est}}= C_{fx} + b_{1}m + b_{2}A + b_{3}m^2 + b_{4}A^2+b_{5}mA \end{aligned}$$

34 $$\begin{aligned} C_{fz_{est}}=C_{fz}+c_{1}Nr+c_{2}A+c_{3}Nr^2+c_{4}A^2+c_{5}NrA\end{aligned}$$

35 $$\begin{aligned} Nu_{est}=Nu+a_{1}Nt+a_{2}Nb+a_{3}Nt^2+a_{4}Nb^2+a_{5}NtNb\end{aligned}$$

36 $$\begin{aligned} Sh_{est}=Sh+d_{1}Nt+d_{2}Nb+d_{3}A+d_{4}Nb^2+d_{5}NtNb+d_{6}Nt^2+d_{7}A^2+d_{8}ANt+d_{9}ANb \end{aligned}$$

where $b_{1},\;b_{2},\;b_{3},\;b_{4},\;b_{5}\;\text {and}\;c_{1},\; c_{2},,\; c_{3},\;c_{4},\;c_{5}$ are the regression coefficients of the quadratic multiple regression model for $C_{fx}Re^{1/2}_{x}\;\text {and}\;C_{fz}Re_{x}^{1/2}$,respectively, while $a_{1},\;a_{2},\;a_{3},\;a_{4},\;a_{5}\;\text {and}\;d_{1},\;d_{2},\;d_{3},\;d_{4},\;d_{5},d_{6},\;d_{7},\;d_{8}$ are indicated the regression coefficients of the quadratic multiple regression model for the reduced Nusselt number $Nu_{x}Re^{-1/2}_{x}$ and the reduced Sherwood number $Sh_{x}Re^{-1/2}_{x}$, respectively. For carrying out the quadratic multiple regression model on the skin friction coefficient in *x*-direction, the values of $C_{fx}Re^{1/2}_{x}$ are estimated for 100 sets of values of $m\;\text {and}\;A$ generated arbitrarily from the intervals $\left[ 0, 0.3\right] \;\text {and}\;\left[ 0,0.15\right]$, respectively, assuming the other parameters as fixed, whereas, in order to perform the same model on the skin friction coefficient in *z*-direction, the numerical values of $C_{fx}Re^{1/2}_{z}$ are approximated for 100 sets of values of $Nr\;\text {and}\;A$ taken randomly from the intervals $\left[ 0.1,0.5\right] \;\text {and}\;\left[ 0,0.15\right]$ keeping the other parameters as constant. Moreover, to execute the quadratic multiple regression model on the reduced Nusselt number, the numerical values of $Nu_{x}Re^{-1/2}_{x}$ are calculated for 100 sets of values of $Nt\;\text {and}\;Nb$ chosen randomly from the intervals $\left[ 0.1,0.5\right] \;\text {and}\;\left[ 0.1,0.5\right]$ assuming the other parameters as constant, whereas for constructing the same model on the reduced Sherwood number, the numerical values of $Sh_{x}Re^{-1/2}_{x}$ are computed for 100 sets of values of $Nt,\;Nb\;\text {and}\;A$ generated arbitrarily from the intervals $\left[ 0.1,0.5\right] ,\;\left[ 0.1,0.5\right] \;\text {and}\;\left[ 0,0.15\right]$, when the numerical values of the other parameters are considered to be constant. The maximum relative errors $\varepsilon _{C_{fx}},\;\varepsilon _{C_{fz}},\;\varepsilon _{Nu}\;\text {and}\;\varepsilon _{Sh}$ are formulated as

37 $$\begin{aligned} \varepsilon _{C_{fx}} = \left|\frac{C_{fx_{est}}-C_{fx}}{C_{fx}}\right|\end{aligned}$$

38 $$\begin{aligned} \varepsilon _{C_{fz}} = \left|\frac{C_{fz_{est}}-C_{fz}}{C_{fz}}\right|\end{aligned}$$

39 $$\begin{aligned} \varepsilon _{Nu} = \left|\frac{Nu_{est}-Nu}{Nu}\right|\end{aligned}$$

40 $$\begin{aligned} \varepsilon _{Sh} = \left|\frac{Sh_{est}-Sh}{Sh}\right|. \end{aligned}$$

From Table 5, it can be notified that with increasing the impact of the Schmidt number, $C_{fx}$ and the values of the coefficient of *A* are negative, while the values of the coefficient of *m* are positive. Consequently, it can be concluded from the Eq. (33) that the favourable nature of the unsteadiness parameter on the magnitude of the skin friction coefficient in *x*-direction is noticed, on the contrary, the reverse trend of Hall Current parameter towards it takes place. Moreover, it can be noted that on increasing the Schmidt number, the values of the coefficient of the unsteadiness parameter are greater than the values of the coefficient of the Hall current parameter in magnitude, which signifies that a small change in the unsteadiness parameter leads to a large perturbation in $C_{fx}Re^{1/2}_{x}$ as compared to the Hall current parameter. Table 6 displays that the values of the coefficient of the unsteadiness parameter are larger than the values of the coefficient of the thermal radiation parameter in magnitude with rising the Hall current parameter, which indicates that the impact of the unsteadiness parameter on $C_{fz}Re^{1/2}_{z}$ is dominant as compared to the thermal radiation parameter. Since the coefficients of $Nr\;\text {and}\;A$ are negative and positive, respectively, from the Eq. (34) it can be clarified that the thermal radiation parameter has a decreasing tendency towards the skin friction coefficient in *z*-direction, whereas the assisting behaviour of the unsteadiness parameter on it can be noted. Table 7 exhibits that the values of the coefficient of the Brownian motion parameter are greater than the values of the coefficient of the thermophoretic parameter in magnitude with a variation in the Prandtl number, which signifies that a slight variation in the Brownian motion parameter causes a substantial change in the reduced Nusselt number $Nu_{x}Re^{-1/2}_{x}$ as compared to the Thermophoretic parameter. However, it can be visualized from the Table 7 that the coefficients of the thermophoretic parameter and Brownian motion parameter become negative with a slight variation in the Prandtl number. So from the Eq. (35), it can be concluded that thermophoretic parameter and Brownian motion parameter have adverse effects on the reduced Nusselt number $Nu_{x}Re^{-1/2}_{x}$. Through the Table 8, it can be ascertained that the values of the coefficient of the Brownian motion parameter are greater than the values of the coefficients of the thermophoretic parameter and the unsteadiness parameter in magnitude, which reveals that the influence of the Brownian motion on the reduced Sherwood number $Sh_{x}Re^{-1/2}_{x}$ is more than the effects of thermophoretic diffusion and unsteadiness parameter. Furthermore, it is prominently observed from the Table 8 that the values of the coefficients of thermophoretic parameter and unsteadiness parameter are negative, while the coefficient of the Brownian motion is positive. Therefore, it is quite evident from the Eq. (36) that the Brownian motion has an enhancing impact on the reduced Sherwood number $Sh_{x}Re^{-1/2}_{x}$, whereas the contrary trend of thermophoretic diffusion and unsteadiness parameter towards it takes place. It is an important finding that the accuracy of the quadratic regression estimate for the skin friction coefficient in *z*-direction is much better than that of the quadratic regression estimates for the skin friction coefficient in *x*-direction, the reduced Nusselt number and the reduced Sherwood number.

**Table 3:** Comparison of numerical values of $Nu_{x}Re_{x}^{\frac{-1}{2}}\;\text {and}\;Sh_{x}Re_{x}^{\frac{-1}{2}}$ against *Nt* for $\beta \rightarrow \infty , Pr=10, Sc=10, Nb=0.1, M=m=Ec=\lambda =\alpha =A=Nr=0$.

Present results			Khan and Pop^3^	
*Nt*	$Nu_{x}Re_{x}^{\frac{-1}{2}}$	$Sh_{x}Re_{x}^{\frac{-1}{2}}$	$Nu_{x}Re_{x}^{\frac{-1}{2}}$	$Nu_{x}Re_{x}^{\frac{-1}{2}}$
0.1	0.9524	2.1294	0.9524	2.1294
0.2	0.6932	2.2740	0.6932	2.2740
0.3	0.5201	2.5286	0.5201	2.5286
0.4	0.4026	2.7952	0.4026	2.7952
0.5	0.3211	3.03511	0.3211	3.03511

**Table 4:** Comparison of numerical values of magnitude of skin friction coefficient in *x*-direction against $M, \beta$ for $m=\lambda =Nr=A=0$.

*M*	$\beta$	Present $-\left( 1+\frac{1}{\beta }\right) f^{''}(0)$	Nadeem et al.^34^ $-\left( 1+\frac{1}{\beta }\right) f^{''}(0)$
0	$\infty$	1.0000	1.0042
	5	1.0954	1.0954
	1	1.4142	1.4142
10	$\infty$	3.3166	3.3165
	5	3.6332	3.6331
	1	4.6904	4.6904
100	$\infty$	10.0499	10.049
	5	11.0091	11.0091
	1	14.2127	14.2127

**Table 5:** Numerical computations for quadratic regression coefficients and error bound for estimated $C_{fx}Re^{1/2}_{x}$.

*Sc*	$C_{fx}$	$b_{1}$	$b_{2}$	$b_{3}$	$b_{4}$	$b_{5}$	$\varepsilon _{C_{fx}}$
0.8	− 3.3897	0.0144	− 0.4060	0.5140	− 0.7100	1.0820	0.0066
1	− 3.3851	0.0146	− 0.4043	0.5183	− 0.6886	1.0687	0.0068
1.2	− 3.3810	0.0147	− 0.4028	0.5209	− 0.6754	1.0607	0.0069

**Table 6:** Numerical computations for quadratic regression coefficients and error bound for estimated $C_{fz}Re^{1/2}_{x}$.

*m*	$C_{fz}$	$c_{1}$	$c_{2}$	$c_{3}$	$c_{4}$	$c_{5}$	$\varepsilon _{C_{fz}}$
0.05	0.1891	− 0.0019	0.0024	0.0115	0.0880	− 0.0556	5.5649E−04
0.06	0.2267	− 0.0022	0.0028	0.0137	0.1039	− 0.0658	5.6294E−04
0.07	0.2643	− 0.0026	0.0032	0.0158	0.1189	− 0.0755	5.7044E−04

**Table 7:** Numerical computations for quadratic regression coefficients and error bound for estimated $Nu_{x}Re^{-1/2}_{x}$.

*Pr*	*Nu*	$a_{1}$	$a_{2}$	$a_{3}$	$a_{4}$	$a_{5}$	$\varepsilon _{Nu}$
0.05	0.6554	− 0.0013	− 0.0016	0.0000	0.0000	0.0000	0.0022
0.08	0.7418	− 0.0026	− 0.0029	0.0000	0.0000	0.0000	0.0037
0.1	0.8061	− 0.0037	− 0.0039	0.0000	0.0000	0.0000	0.0047

**Table 8:** Numerical computations for quadratic regression coefficients and error bound for estimated $Sh_{x}Re^{-1/2}_{x}$.

*m*	*Sh*	$d_{1}$	$d_{2}$	$d_{3}$	$d_{4}$	$d_{5}$	$d_{6}$	$d_{7}$	$d_{8}$	$d_{9}$	$\varepsilon _{Sh}$
0.1	1.1996	− 0.0430	0.0620	− 0.0519	0.0977	0.2736	0.0543	1.9794	− 0.7276	− 1.3769	0.0049
0.3	1.1996	− 0.0438	0.0623	− 0.0505	0.0987	0.2760	0.0545	1.9940	− 0.7331	− 1.3882	0.0049
0.5	1.1997	− 0.0450	0.0629	− 0.0483	0.1005	0.2800	0.0548	2.0177	− 0.7422	− 1.4064	0.0049

## Conclusions

Our main objective of carrying out the present research work is to analyze the features of entropy generation on unsteady three dimensional magnetohydrodynamic flow of Casson nanofluid over the stretching sheet under the influence of radiative heat transfer, mixed convection, Hall current, thermophoresis, Brownian motion, Ohmic heating and heat generation. The governing boundary layer equations consisting of nonlinear coupled partial differential equations are transformed into ordinary differential equations by using Similarity transformation variables. The resulting highly nonlinear coupled ordinary differential equations are solved numerically by utilizing the spectral quasi-linearization method and Chebyshev spectral collocation method. The physical impact of several flow parameters are exhibited through various graphs and tables. The key observations of the present investigation are summarized as follows:

- Magnetic parameter and Casson parameter lead to retard the Casson nanofluid motion in *x*- direction, which results in decreasing the momentum boundary layer thickness. But in case of transverse velocity, the dual nature of these parameters is observed prominently. On increasing the magnetic parameter and Casson parameter, the transverse velocity is decreasing drastically away from the sheet, but the reverse phenomena is noticed in the region closer to the stretching sheet. On the contrary, the magnetic parameter has a tendency to increase temperature profile.
- The Unsteadiness parameter has no considerable effect on the velocity distribution of Casson nanofluid. Hall current parameter leads to enhance velocity in *x*- direction slowly, whereas it boosts to increase the transverse velocity highly.On the other hand, Hall current parameter declines temperature, which leads to decay the thermal boundary layer thickness. Moreover, Eckert number and mixed convection parameter increase the momentum of Casson nanofluid strictly. As a result of which, the increment of momentum boundary layer thickness is witnessed. However, the increasing trend of mixed convection parameter towards the temperature distribution is noticed significantly.
- In the absence of viscous and Joule dissipations ($Ec=0$), Casson parameter has a favourable effect on temperature profile. On the contrary, it is noticeable that Casson parameter has a tendency to decay temperature in the presence of viscous and Joule dissipations ($Ec>0$). However, the increment in the strength of viscous and Joule dissipations, Brownian motion, thermophoretic diffusion, heat generation results in an increment of temperature distribution, which indicates that the thermal boundary layer becomes thicker. Adjacent to the sheet, in case of temperature distribution, the radiation parameter acts as an assisting parameter, while far from the plate, it acts as an opposing parameter.
- Brownian motion is responsible to diminish the species concentration throughout the boundary layer, whereas thermophoretic diffusion has an opposing trend towards the species concentration.
- The magnitude of skin friction coefficient in *x*-direction gets decreased due to increasing the parameters $A,\; m,\;\beta ,\; Nr, \;tr,\; Ec,\; \lambda ,\; \alpha ,\; Nt,\; Nb\;\text {and}\; Sc$, while the increasing effect of the parameters $M\;\text {and}\;Pr$ is visualized on it. The magnitude of skin friction coefficient in *z*-direction is an increasing function of parameters $A,\; M,\; m,\; Nr, \;tr,\; Ec,\; \lambda ,\; \alpha ,\; Nt,\; Nb\;\text {and}\;Sc$, whereas the parameters $\beta \;\text {and}\;Pr$ have a reverse effect on it. The parameters $A,\; M,\; Ec,\;Pr,\; \lambda ,\; \alpha ,\; Nt,\; Nb\;\text {and}\; Sc$ have an increasing trend to the rate of heat transfer in magnitude, but the parameters $m,\;\beta ,\;Nr,\;tr$ have an adverse effect on it. On the other hand, the parameters $A,\; M,\; Ec,\;Pr,\; \lambda ,\; \alpha ,\; Nt,\;\text {and}\; Sc$ have a tendency to enhance the rate of mass transfer in magnitude, while the parameters $m,\;\beta ,\;Nr,\;tr \text {and}\;Nb$ have an opposing effect on it.
- The entropy generation is inferred to rise for increasing the diffusive variable, concentration ratio parameter and Brinkman number, whereas these parameters have an opposing effect on Bejan number. The Magnetic parameter diminishes entropy generation closer to the sheet and enhances the entropy generation drastically away from the sheet. The similar phenomena can be noticed in case of Bejan number. Moreover, the opposite observation can be visualized in case of entropy generation and Bejan number against the Hall current parameter. Casson parameter decreases the entropy generation, while its adverse trend is observed to the Bejan number.
- The increment in the strength of thermal radiation is highly responsible for the larger entropy generation and Bejan number.
